# Zebrafish Models for Human Acute Organophosphorus Poisoning

**DOI:** 10.1038/srep15591

**Published:** 2015-10-22

**Authors:** Melissa Faria, Natàlia Garcia-Reyero, Francesc Padrós, Patrick J. Babin, David Sebastián, Jérôme Cachot, Eva Prats, Mark Arick II, Eduardo Rial, Anja Knoll-Gellida, Guilaine Mathieu, Florane Le Bihanic, B. Lynn Escalon, Antonio Zorzano, Amadeu M.V.M Soares, Demetrio Raldúa

**Affiliations:** 1 Department of Biology and CESAM, University of Aveiro, Portugal; 2 IDÆA-CSIC, Jordi Girona 18, 08034, Barcelona, Spain; 3 Environmental Laboratory, US Army Engineer Research and Development Center, Vicksburg, MS, USA; 4 Institute for Genomics, Biocomputing & Biotechnology (IGBB), Mississippi State University, Starkville, Mississippi, USA; 5 Pathological Diagnostic Service in Fish, Universitat Autònoma de Barcelona, 08190 Bellaterra, Spain; 6 Rare Diseases, Genetic and Metabolism (MRGM), Université de Bordeaux, EA 4576, F-3340 Talence, France; 7 Institute for Research in Biomedicine (IRB Barcelona), 08028 Barcelona, Spain; 8 Departament de Bioquímica i Biologia Molecular, Facultat de Biologia, Universitat de Barcelona, 08028 Barcelona, Spain; 9 Instituto de Salud Carlos III, Centro de Investigación Biomédica en Red de Diabetes y Enfermedades Metabólicas Asociadas (CIBERDEM), 08017 Barcelona, Spain; 10 EPOC, UMR CNRS 5805, Université de Bordeaux, 33405 Talence, France;; 11 CID-CSIC, Jordi Girona 18, 08034, Barcelona, Spain; 12 Department of Cellular and Molecular Medicine, CIB-CSIC, Ramiro de Maetzu 9, 28040, Madrid, Spain

## Abstract

Terrorist use of organophosphorus-based nerve agents and toxic industrial chemicals against civilian populations constitutes a real threat, as demonstrated by the terrorist attacks in Japan in the 1990 s or, even more recently, in the Syrian civil war. Thus, development of more effective countermeasures against acute organophosphorus poisoning is urgently needed. Here, we have generated and validated zebrafish models for mild, moderate and severe acute organophosphorus poisoning by exposing zebrafish larvae to different concentrations of the prototypic organophosphorus compound chlorpyrifos-oxon. Our results show that zebrafish models mimic most of the pathophysiological mechanisms behind this toxidrome in humans, including acetylcholinesterase inhibition, N-methyl-D-aspartate receptor activation, and calcium dysregulation as well as inflammatory and immune responses. The suitability of the zebrafish larvae to *in vivo* high-throughput screenings of small molecule libraries makes these models a valuable tool for identifying new drugs for multifunctional drug therapy against acute organophosphorus poisoning.

Organophosphorus (OP) compounds are a class of acetylcholinesterase (AChE) inhibitors used as not only pesticides but also chemical warfare nerve agents. Epidemiological studies regarding OP pesticides estimate approximately 3 million cases of acute severe poisoning and 300,000 deaths annually, most of them in developing countries of the Asia-Pacific region^1^. In contrast, developed nations are most concerned about the potential military and terrorist use of these compounds because troops or civil populations can be exposed to not only chemical warfare agents based on these groups of compounds, such as soman, sarin, tabun and VX, but also pesticides or industrial OPs if they are used as improvised or dirty chemical weapons in asymmetric warfare or terrorism. Thus, acute OP poisoning (OPP) is a major clinical and public health problem. The inhibition of AChE by OP compounds leads to accumulation of the neurotransmitter acetylcholine (ACh) at the cholinergic synaptic clefts, with the consequent long-term activation of the nicotinic and muscarinic ACh receptors (AChR) and overstimulation of cholinergic neurons as well as hyperexcitation and seizures^2^. Following the initial cholinergic overstimulation, a cascade of downstream events occurs that leads to secondary neuronal and muscle toxicity. Thus, the onset of OP-induced seizures allows the release of excitatory amino acids (EAAs) such as glutamate and aspartate, which activate N-methyl-D-aspartate (NMDA) receptors, resulting in an intracellular influx of Ca^2+^. Shortly following OP poisoning, released EAAs can maintain the seizures independent of the initial cholinergic overstimulation^3^. OP-evoked seizures can progress to *status epilepticus* and severe brain damage^4^,^5^. Excessive accumulation of intracellular Ca^2+^ can activate different lipases, proteases, endonucleases, kinases or phosphatases, resulting in damage to cell membranes, cytoskeleton or organelles^6^. Oxidative stress, with reactive oxygen and nitrogen species (ROS and RNS, respectively) generation, also plays an important role in the neuroinflammation and cellular death found in OPP^2^,^7^. Although many different mechanisms are involved in the pathophysiology of OPP, standard therapy only targets a few of them, such as muscarinic receptor antagonists (atropine), cholinesterase reactivators (oximes) and anticonvulsants (benzodiazepines), although the results from different clinical studies are inconclusive regarding the efficacy of oximes and benzodiazepines in reducing morbidity or mortality in humans^8^,^9^. The World Health Organization has noted the necessity of introducing newer, more effective antidotes to improve the results of classical treatments^10^. Recently, multifunctional drug therapies including not only the standard treatment but also antioxidants, calcium influx blockers and NMDA antagonist have been proposed to prevent secondary neurological damage^6^.

Development and validation of *in vivo* animal models for rapid screening of molecular libraries to identify potential medical countermeasures against anticholinesterase agents is one of the priorities of the National Institutes of Health CounterACT programme^11^. An animal model suitable for use in the identification of new drugs for OPP treatment should recapitulate the most relevant pathophysiological mechanisms in humans. Most models for OPP developed thus far use rodents^12^, and although these models have been extremely useful for deciphering the pathophysiological mechanisms behind OPP, models built in rodents are not suitable for *in vivo* high-throughput screening of chemical libraries. Zebrafish (*Danio rerio*) is an animal model increasingly used in biomedical research including human toxicology^13^,^14^. One key advantage of zebrafish embryos/larvae over other vertebrate models for drug discovery is their suitability for *in vivo* high-throughput screening of chemical libraries for pharmacological and/or toxicological effects^15^. Different data support the use of zebrafish for the development of new animal models of OPP. First, zebrafish AChE expression and catalytic properties have already been well characterized^16^. Moreover, using the *ache* mutant, in which AChE activity is completely abolished, non-acetylcholinesterase secondary targets of different AChE inhibitors have been identified^17^. Although the developmental neurotoxicity of OP compounds has been recently characterized in zebrafish embryos, the clinical features and pathophysiological mechanisms involved in OPP in zebrafish remain to be characterized^18^,^19^.

In this study, we developed and characterized zebrafish models for mild, moderate and severe OPP by acute exposure of zebrafish to different concentrations of the prototypic OP compound chlorpyrifos-oxon (CPO). Pathophysiological processes involved in the development of OPP in zebrafish were characterized at different levels of organization, from the whole-organism level to the molecular level. Our results showed that zebrafish models for OPP mimic most of the aspects of human OPP, indicating that zebrafish is a suitable model for identifying new antidotes against OPP.

## Results and Discussion

### Grading severity of chlorpyrifos-oxon poisoning in zebrafish larvae

**Figure 1:**
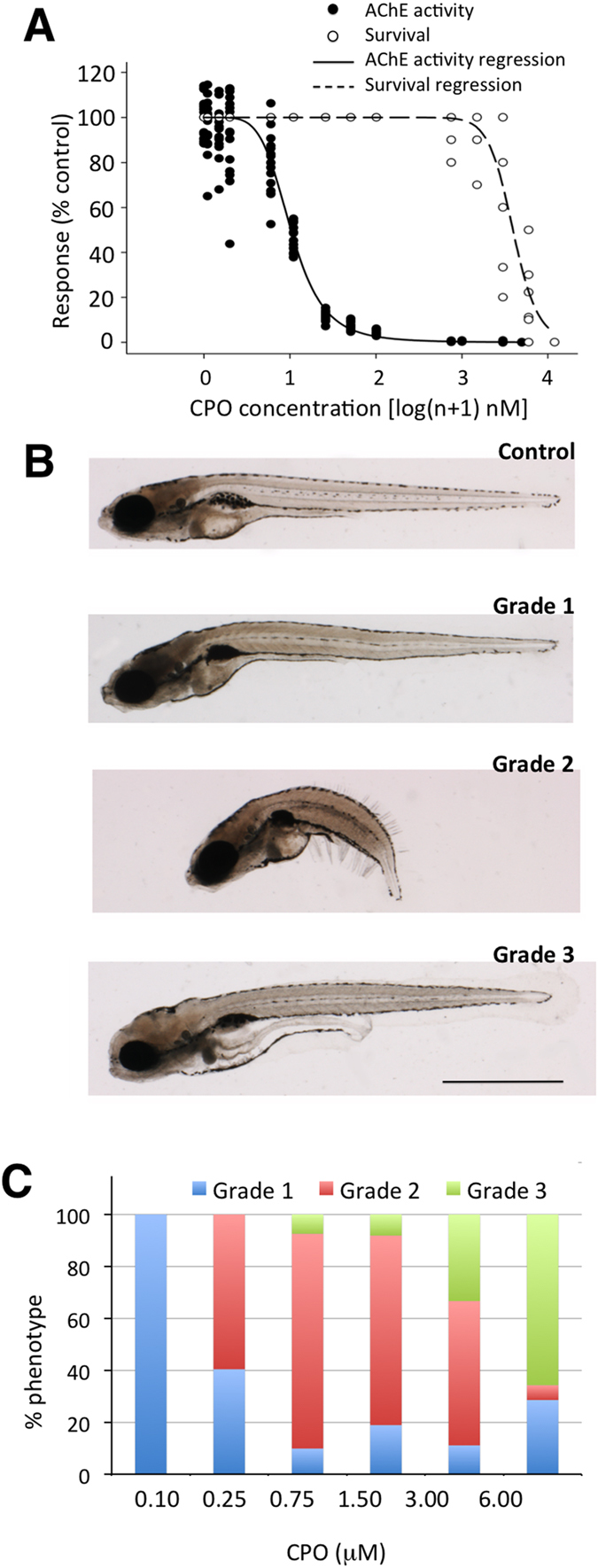
Chlorpyrifos-oxon (CPO) induces concentration-dependent inhibition of acetylcholinesterase (AChE) and the expression of three different phenotypes in zebrafish larvae. (**A**) Concentration-response analysis of CPO regarding the inhibition of AChE activity and survival in zebrafish larvae exposed to different CPO concentrations from 7 to 8 days post fertilization (24 h exposure, 16 larvae per experimental group, p < 0.0001; AChE activity, *F*(1,143) = 1950.77, *r*^*2*^ = 0.978; survival, *F*(1,59) = 251.295, *r*^*2*^ = 0.9208). (**B**) Lateral views of representative 8 days post fertilization larvae control and the mild (grade 1), moderate (grade 2) and severe (grade 3) phenotypes. (**C**) Prevalence of the three different phenotypes in response to different CPO concentrations (48 larvae per experimental group). Scale bar: 1 mm.

OPP can be graded by severity as mild, moderate and severe^20^. To develop zebrafish models for the different grades of OPP, 7 days post fertilization (dpf) larvae were exposed to different concentrations of CPO, the active metabolite of chlorpyrifos and a prototypic OP compound, for 24 h. The measured concentrations of CPO in fish water at the beginning of the exposure periods were very close to the nominal values and decreased significantly after 24 h incubation with larvae (Supplementary Table 1). A strong inhibitory effect of CPO on AChE activity was found as early as 1 h after exposure, with a 50% inhibitory concentration (IC50) of 64.34 ± 1.44 nM CPO (Supplementary Fig. S1). When concentration-response analysis of AChE activity (Fig. 1A) was performed after 24 h of exposure, the IC50 decreased to 9.58 ± 0.35 nM. Interestingly, zebrafish larvae showed high resistance to CPO, with a 24 h−50% lethal concentration (24 h LC50) more than one order of magnitude higher than adults (3.97 ± 0.14 vs 0.13 ± 0.01 μΜ CPO for larvae and adults, respectively), and no significant mortality was found at AChE activities higher than 1% of the control values (Fig. 1A, Supplementary Fig. S2 and Supplementary Discussion). The high resistance of larvae to death by CPO allowed the expression of different phenotypes partially related to the CPO concentration (Fig. 1B,C). We graded the severity of the CPO poisoning in zebrafish larvae in three primary phenotypes according to morphological and behavioural criteria. The mildest (grade 1) phenotype was characterized by moderate reduction in the locomotor activity with no or only mild defects in gross morphology (Fig. 1B). All larvae exposed to 100 nM CPO exhibited a grade 1 phenotype (Fig. 1C), and the mean AChE activity at this concentration was 4.18% of the control. The moderate (grade 2) phenotype was primarily characterized by complete paralysis of axial muscles and a dramatic decrease in the trunk length (Fig. 1B and Supplementary Fig. S3A). The highest prevalence of the grade 2 phenotype was found in larvae exposed to 0.75–1.50 μΜ CPO, with mean AChE activity of 0.13% of the control (Fig. 1A,C). Interestingly, despite the striking morphological changes observed in the grade 2 phenotype, a partial recovery of both the phenotype and AChE activity was found 3 days after transferring the larvae to clean water (Supplementary Fig. S4). Finally, the most severe (grade 3) phenotype was characterized by complete paralysis of axial muscles and altered morphology of the head, and the highest prevalence of this phenotype was found in larvae exposed to 3–6  μΜ CPO (Fig. 1B,C and Supplementary Fig. S3), with mean AChE activity of only 0.024% of the control. In contrast to the grade 2 phenotype, no rescue of morphology was found when full grade 3 larvae were transferred to clean water, and 100% lethality occurred within the next 24 h, indicating that this phenotype represents an irreversible and lethal condition. Unless stated otherwise, grade 1, 2 and 3 larvae used for phenotypic characterization and pathophysiological mechanism analysis were generated using 0.1, 1.0 and 3.0 μΜ CPO, respectively.

### Grade 1 larvae exhibit behavioural impairment that correlates with AChE inhibition

**Figure 2:**
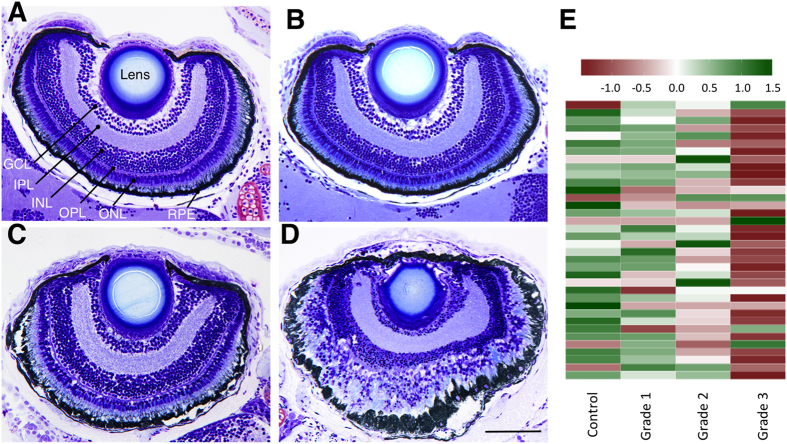
CPO induces concentration-dependent impairment of the retina architecture and the phototransduction pathways in zebrafish larvae. Retinal histology (transverse plastic semithin sections) of representative control (**A**), grade 1 (**B**) grade 2 (**C**) and grade 3 (**D**) 8 days post fertilization zebrafish larvae shows a good relationship between the grade of the phenotype and the severity of the effects on the retinal architecture. (**E**) Heatmap of the phototransduction pathway (dre04744) in zebrafish control and grades 1, 2 and 3 larvae, showing a clear relationship between the grade of phenotype severity and the degree of down-regulation of this pathway. Abbreviations: *GCL*, ganglion cell layer; *INL*, inner nuclear layer; *IPL*, inner plexiform layer; *ONL*, outer nuclear layer; *OPL*; outer plexiform layer; *RPE*, retinal pigment epithelium. Scale bar: 100 μm.

Grade 1 was the most prevalent phenotype at low CPO concentrations. No morphological defects were found in larvae exposed to concentrations below 100 nM, although 22% (n = 58) of the larvae exhibited mild tail curvature at this concentration. Morphometric analysis showed a mild but significant reduction in the length of the trunk compared to the untreated controls, and a parallel decrease in the length of the axial slow muscle fibres was found (Supplementary Fig. S5C,D). Histopathological assessment failed to identify any effect on the central and peripheral nervous system, retina or axial muscle fibres (Fig. 2A,B and Supplementary Figs S6 and S7A,B).

**Figure 3:**
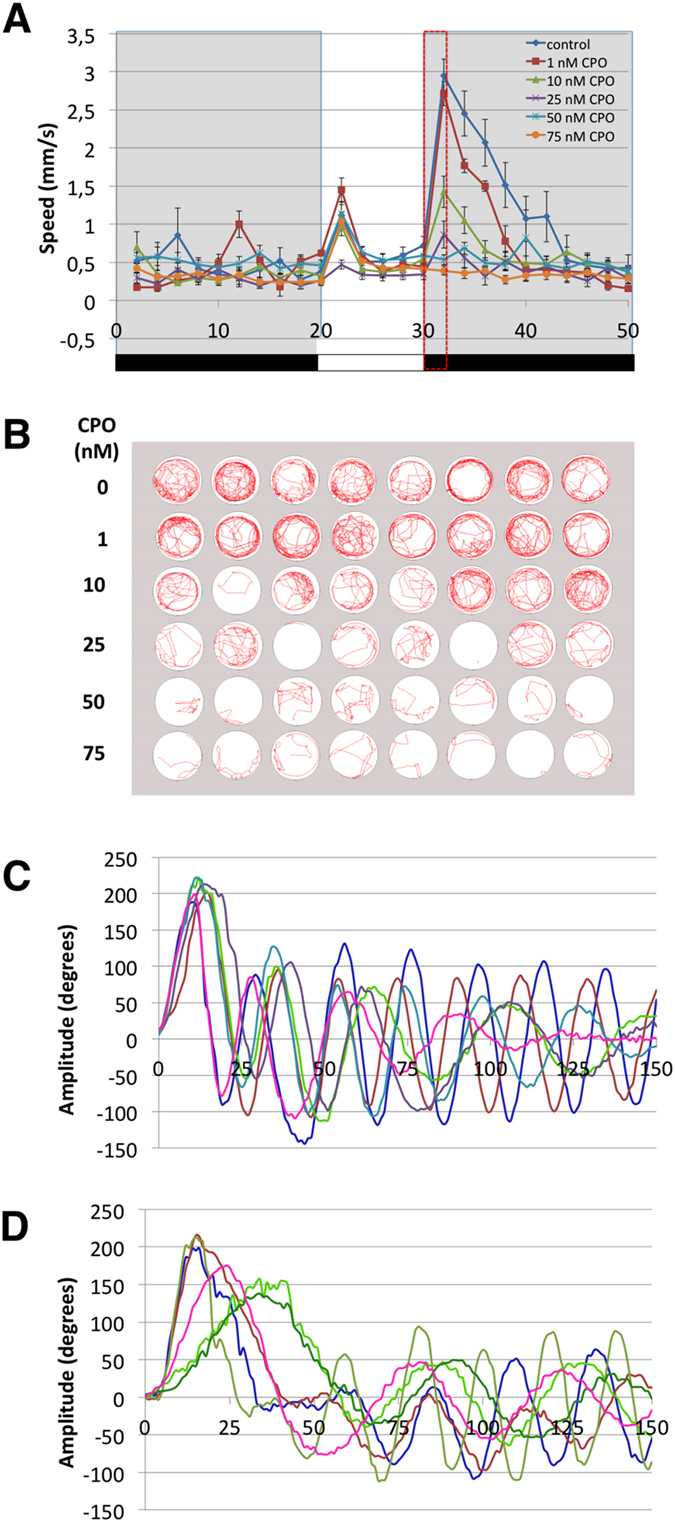
Motor behaviour is strongly impaired by chlorpyrifos-oxon in zebrafish larvae. (**A**) VMR profiles, showing that the hyperactivity peak evoked in response to sudden exposure to darkness is reduced until total abolition when 8 days post fertilization zebrafish larvae were previously exposed to increasing concentrations of CPO for 24 h. (**B**) Locomotion tracking plots for the VMR assay in a 48-well behavioural arena (1 larva per well) recorded a decrease in the swimming activity with increasing chlorpyrifos-oxon concentrations during the first 2 min of the light/dark challenge (30–32 min of the assay). (**C,D**) Kinematics of TMR traces for control (**C**) and grade 1 (**D**) larvae. For each condition, six representative traces are shown from the first 150 ms of the escape response. Each trace is from a different larva. The curvature of the body is represented in degrees, with 0 indicating a straight body. Only grade 1 larvae without any morphological defects were selected for behavioural analyses.

Altered motor behaviour is one of the most sensitive endpoints in OPP by chlorpyrifos in rats^21^. Thus, motor behaviour was analysed in grade 1, the only grade with mobile larvae, using a battery of behavioural tests including basal locomotor activity, visual motor response (VMR) and touch-evoked escape response (TMR). Basal locomotor activity was analysed by measuring the distance moved by the larvae in 20 min. A significant reduction in basal motor activity was found in grade 1 larvae exposed to 10, 50 and 100 nM CPO compared to the control (Supplementary Fig. S8). VMR, a transient period of hyperactivity exhibited by zebrafish larvae in response to sudden decreases in light intensity^22^, was evaluated in grade 1 by tracking movement in response to light-dark transition. Figure 3A,B show a clear and concentration-dependent decrease in the VMR starting at 10 nM CPO and reaching the total abolition of this behaviour from 50 nM CPO onwards. When the AChE activity was measured on the same set of larvae used for the behavioural analysis, a significant correlation was found between VMR and AChE activity (Supplementary Fig. S9). Thus, the IC50 value for AChE activity and the 50% effective concentration (EC50) for VMR were 9.58 and 8.42 nM CPO, respectively, and the non-observed effect concentration (NOEC) for both endpoints was 1 nM CPO. VMR integrates sensory and motor responses. Thus, changes in illumination are detected by the OFF streams in the larvae retina and by deep brain photoreceptors, whereas specific motor circuits are involved in the evoked motor response^22^,^23^,^24^. To understand the component of the VMR altered in grade 1 larvae, the kinematics of TMR, a highly stereotyped motor behaviour evoked by non-visual stimuli, was analysed^23^. TMR is a complex behaviour constituted by three sequential modules of single behaviour, (1) a very fast and large C-bend followed by (2) a high amplitude counterbend and (3) a bout of fast swimming oriented away from the stimuli^23^. Whereas kinematic analysis of TMR in control larvae exhibited the stereotyped profile, all grade 1 larvae analysed exhibited some abnormalities in the escape response, including a slower and/or lower amplitude C-bend, an altered counterbend and decreased frequency and amplitude of the last module (Fig. 3C,D). Even considering that grade 1 larvae exhibit an altered response to the touch, the fact that a non-visual stimulus is able to elicit a motor response supports the hypothesis that the total abolition of the VMR found in grade 1 larvae is produced by a specific action of CPO on the visual function.

Aiming to understand the molecular phenotype of the different grades of OPP identified in zebrafish, a large-scale transcriptomic analysis was performed using an RNA-seq approach. In total, 80 differentially expressed genes (DEGs) were found in grade 1 larvae (FDR-adjusted p ≤ 0.05; Supplementary Data 1). When the significant perturbed KEGG pathways were analysed, four pathways were down-regulated (FDR-adjusted p ≤ 0.05), and none was significantly up-regulated (Supplementary Data 2). Interestingly, phototransduction (dre04744) was one of the down-regulated pathways, a result supporting the hypothesis that the dramatic effect on the VMR found in grade 1 larvae is mediated by a specific action of CPO on the visual function.

**Figure 4:**
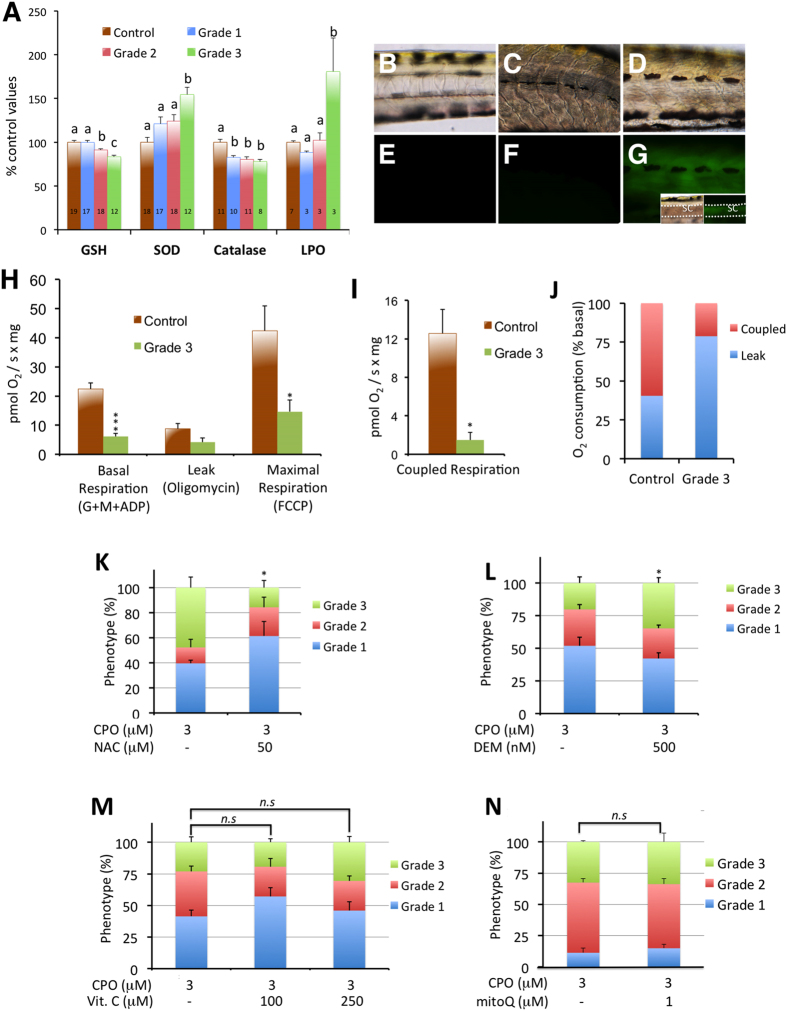
Oxidative stress is only induced in grade 3 larvae. (**A**) SOD and catalase activities and GSH and MDA (LPO) levels in control and grade 1–3 zebrafish larvae exposed from 7 to 8 days post fertilization (dpf). The data represent the mean ± SEM. The value on each bar indicates the number of pools for each condition, and bars with different letters are significantly different (p < 0.05, one-way ANOVA with Tukey’s multiple comparison test). (**B**–**G**) Brightfield (**B–D**) and fluorescence (**E–G**) images of the trunks of representative 8 dpf control (**B,E**), grade 2 (**C,F**), and grade 3 (**D,G**) larvae phenotypes. The oxidative fluorescent dye DCFH-DA, which is used for identifying ROS generation in live larvae, showed high ROS generation in grade 3 muscle fibres and spinal cord (**G**) compared to grade 2 (**F**) and control (**E**). Inset at (**G**) shows brightfield and fluorescence images of the trunk of a grade 3 larva, with the focal plane at the level of the spinal cord. Dorsal and ventral limits of the spinal cord are highlighted by a white dotted line. Abbreviations: sc, spinal cord. (**H**–**J**) Grade 3 larvae exhibit a significant reduction in mitochondrial respiration. The basal and maximal respiration (**H**) and the coupled respiration (**I**) were strongly reduced in grade 3 larvae with respect to the control larvae (3–11 pools with 20–25 larvae each were analysed for each group; *p < 0.05, ***p < 0.001, Student’s *t*-test). Moreover, the ratio between coupled respiration and leak was strongly altered (**J**). (**K,L**) Prevalence of the grade 3 phenotype can be modified by altering the endogenous levels of GSH. Larvae were pre-incubated with either 50 μM N-acetyl-L-cysteine (NAC) or 0.5 μM diethylmaleate (DEM) for 24 h (from 6 to 7 dpf) followed by co-exposure with 3 μΜ CPO for 24 h (from 7 to 8 dpf). (**K**) Increasing the GSH levels with N-acetyl-L-cysteine (NAC) decreased the prevalence of the grade 3 phenotype by 67% [3 groups with 8–19 larvae each were analysed for 3 μΜ CPO and 3 groups with 13–23 larvae for 3 μΜ CPO + 50 μΜ NAC; p < 0.05, Student’s *t*-test]. (**L**) Decreasing the GSH levels with diethylmaleate (DEM) increased the prevalence of grade 3 larvae by 82% [6 groups with 22–46 larvae each were analysed for 3 μΜ CPO and 6 groups with 21–43 larvae for 3 μΜ CPO + 0.5 μΜ DEM; p < 0.05, Student’s *t*-test]. (**M,N**) Prevalence of the grade 3 phenotype in zebrafish larvae exposed to 3 μM CPO is not significantly reduced by either antioxidant. Larvae were pre-treated with the antioxidants vitamin C and MitoQ for 3 h or 24 h, respectively, followed by co-exposure with 3 μM CPO for an additional 24 h (**M**); 4 groups with 13–22 larvae each per condition; p > 0.05, one-way ANOVA with Dunnett’s multiple comparison test) or MitoQ (**N**); 4–8 pools with 19–24 larvae each per condition; p > 0.05, Student’s *t*-test). Abbreviations: *n.s*., not significant.

Some evidence suggests that in addition to cholinergic dysregulation, exposure to OP compounds may result in oxidative stress by an imbalance between the generation of radical oxygen species (ROS) and the antioxidant defences^2^. When the presence of oxidative stress was analysed in grade 1 larvae by measuring the status of the antioxidant defence system and lipid peroxidation, catalase was the only member of the antioxidant system with activity significantly different from control values, while lipid peroxidation levels were indistinguishable from control levels (Fig. 4A). These results demonstrate that oxidative stress is not a relevant mode of action for grade 1 OPP. Therefore, the significant correlation found between AChE activity and visual behaviour, along with the absence of lipid peroxidation, strongly indicates that inhibition of AChE, but not oxidative stress, is the primary key event in the pathophysiology of CPO toxicity in grade 1 larvae.

### Grade 2 larvae exhibit hypercontracture of axial muscle fibres

**Figure 5:**
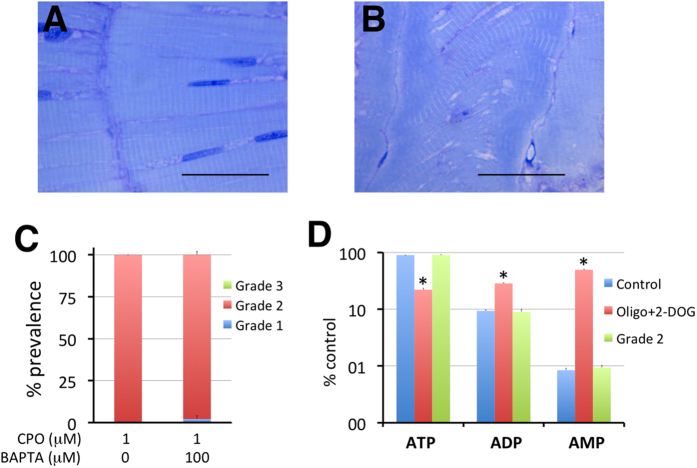
Hypercontracture of the axial muscles in grade 2 larvae is not related with calcium overload or ATP depletion. (**A**,**B**) Morphology and distribution of fast-twitch fibres in the trunks of representative control (**A**) and grade 2 (**B**) larvae. Notice the regular distribution of the fibres, position of the nuclei, negligible spaces between fibres, and homogeneous distribution of the sarcomeres into the individual fibres in control larvae (**A**). In contrast, grade 2 larvae (**B**) present heavily altered myomeric structures, with clear shortening of the myomeres and heterogeneous alignment of the fibres and white spaces. A dense blue band is also present at the myoseptum level. (**C**) BAPTA-AM, a permeable calcium chelator, is not able to rescue the grade 2 phenotype, indicating that Ca^2 + ^overload is not the mechanism leading to the hypercontracture [3 groups per condition with 16 larvae each group, p = 0.187, Student’s *t*-test (t(4) = 1.225)]. Six days post fertilization (dpf) larvae were pre-treated for 24 h with 100 μM BAPTA-AM and then co-exposed to 100 μM BAPTA-AM/1 μM CPO for an additional 24 h. (**D**) Seven days post fertilization larvae that were pre-treated with 40 mM 2-deoxyglucose (2-DOG) for 24 h and then treated with a cocktail of 5 μM oligomycin/40 mM 2-DOG for an additional 2 h exhibited ATP depletion. In contrast, no differences in the content of adenosine phosphates (ATP, ADP, and AMP) were found between the grade 2 and control larvae. Scale bars: 100 μm.

Grade 2 was the most prevalent phenotype within the 0.75–1.50 μM range of CPO concentrations. The primary morphological feature of this phenotype was the hypercontracture of the axial muscle fibres, with a severe reduction in the length of the trunk. Thus, morphometric analysis showed that the trunk of grade 2 larvae exposed to 0.75 μΜ CPO was 56% shorter than the control values. Measurement of the axial slow-twitch muscle fibre length also showed strong shortening with respect to the control (Supplemental Fig. S5C,D). When integrity and the highly organized alignment of axial muscle fibres of grade 2 larvae were evaluated, although no clear effects on axial slow-twitch fibres were found (Supplemental Fig. S7), both parameters were strongly impaired for the fast-twitch fibres, which are the bulk of the zebrafish axial muscle (Fig. 5A,B). Grade 2 larvae were unable to swim at any concentration tested. At CPO concentrations of 0.75 μΜ or below, the larvae exhibited strong stiffness, and above this concentration, the larvae became completely paralyzed, with the TMR fully abolished (Supplementary Movie 1). Interestingly, at CPO concentrations of 0.75 μΜ and below, some grade 2 larvae exhibited creeping, with increased pectoral fin and jaw movements (Supplemental Movie 2). Histopathological analysis of grade 2 larvae failed to identify clear effects on the CNS (Supplementary Fig. S10). Analysis of the retina (Fig. 2) showed moderate effects on the retinal pigment epithelium (RPE) and on the photoreceptors of the outer nuclear layer (ONL).

RNA-seq analysis of grade 2 larvae identified 4,568 DEGs (Supplementary Data 3). Thirty pathways were down-regulated, including phototransduction (dre04744; Fig. 2E and Supplementary Data 4). In addition to the set of genes included in dre04744, RNA-seq analysis showed the down-regulation of 6 of the 9 cone opsin genes present in zebrafish, including long (red; *opn1lw2*), medium (green; *opn1mw1*, *opn1mw2*, and *opn1mw3*), and short (blue; *opn1sw1* and *opn1sw2*) wavelength-sensitive opsins. These results at the transcriptional level are consistent with the moderate disruption of the photoreceptors at the ONL found in the retina. Forty-one pathways were up-regulated (Supplementary Data 5), including most of those involved in immune and inflammatory responses, such as proteasome (dre03050), toll-like receptor signalling pathway (dre04620), MAPK signalling pathway (dre04010), and RIG-I-like receptor signalling pathway (dre04622). Interestingly, recent experimental evidence has linked OPP to activation of the inflammatory and immune responses in mammals^7^,^25^.

Although the clinical signs of grade 2 larvae were consistent with cholinergic overstimulation, non-acetylcholinesterase secondary targets of CPO^26^ could also play a role in the initiation of this phenotype. To test this hypothesis, the effect of pharmacological reactivation of AChE activity using oximes was analysed. When larvae were co-exposed to 1 μΜ CPO and 200 μΜ pralidoxime (2-PAM), an oxime commonly used in human OPP treatment, partial recovery of AChE activity with a full rescue of the grade 2 phenotype was found (Supplementary Fig. S11), demonstrating that AChE inhibition is an upstream event in the toxic pathways that result in the grade 2 phenotype.

Then, we attempted to understand the pathophysiological mechanisms leading to hypercontracture, the primary clinical feature of the grade 2 phenotype. At the cellular level, hypercontracture might originate from either cytosolic Ca^2+^ overload or a rigor-type mechanism resulting from depletion of ATP levels^27^. On one hand, if Ca^2+^ overload was the key event for the hypercontracture of the axial muscle fibres found in grade 2 larvae, then decreasing cytoplasmic Ca^2+^ levels with a permeable calcium chelator should decrease the prevalence of this effect significantly. To test this hypothesis, larvae were pre-exposed for 24 h (6–7 dpf) to 100 μΜ BAPTA-AM, a permeable calcium chelator, and later co-exposed to 1 μΜ CPO and 100 μΜ BAPTA-AM for an additional 24 h. The co-exposure with the permeable calcium chelator had no effect on the prevalence of the hypercontracted phenotype (Fig. 5C), indicating that the hypercontracture found in grade 2 larvae is not mediated by Ca^2+^ overload. On the other hand, intracellular ATP depletion and altered ATP/ADP and ATP/AMP ratios have been reported in skeletal muscles of rats exposed to OPs^28^; thus, depletion in the ATP levels in the muscle fibres might be involved in the observed hypercontracture. To test the hypothesis that a rigor-type mechanism is involved in the development of hypercontracture of axial muscles in grade 2 larvae, the potential depletion of high-energy phosphate compounds (ATP, ADP, and AMP) was analysed. As a positive control, we blocked ATP production in 8 dpf zebrafish larvae by exposing the larvae to 40 mM 2-deoxyglucose (2-DOG) for 24 h and then to a cocktail of 5 μM oligomycin and 40 mM 2-DOG for an additional 2 h. Whereas larvae treated with the cocktail oligomycin & 2-DOG exhibited ATP content depletion, the ATP content in grade 2 larvae was similar to that of control larvae (Fig. 5D, Supplementary Fig. S12 and Supplementary Table 2). These results demonstrated that the hypercontracture found in grade 2 larvae did not originate from the depletion of ATP levels in the whole animal, although specific bioenergetic problems at the sarcomere level cannot be ruled out (Supplementary Discussion).

When the presence of oxidative stress in the grade 2 larvae was analysed by determining antioxidant defence enzyme activity, ROS generation and lipid peroxidation, only mild modulation of the levels of endogenous GSH and catalase activity was found, with neither ROS generation nor lipid peroxidation (Fig. 4A–G), demonstrating that oxidative stress is not relevant to grade 2 pathophysiology.

### Grade 3 phenotype is characterized by widespread necrosis at the CNS and neuromuscular system

**Figure 6:**
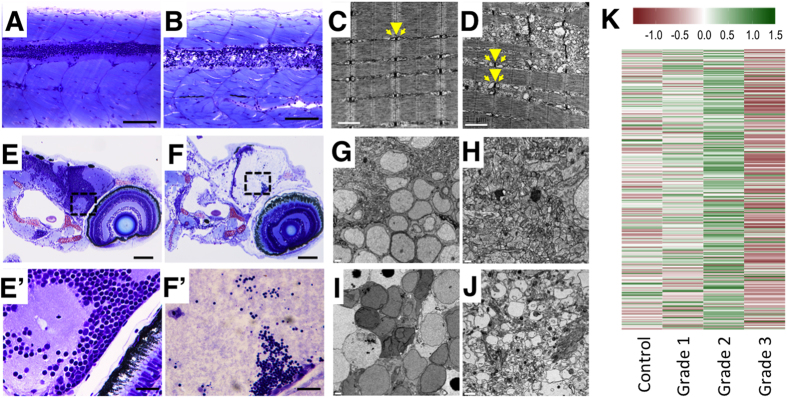
Histopathological assessment of grade 3 larvae shows severe lesions in the central nervous system and muscle fibres. (**A**,**B**) Myotomes with fast-twitch fibres and the spinal cord of representative control (**A**) and grade 3 (**B**) larvae in a medial plane section of the trunk. In the grade 3 larva, severe alterations in the fast-twitch fibres and spinal cord are evident. The spinal cord displays severe necrotic changes, with disruption of the normal nuclear distribution in the neuronal bodies (grey matter) and severe vacuolization of axons (white matter). (**C**,**D**) Electron micrographs of fast-twitch muscle from control (**C**) and grade 3 (**D**) larvae. Whereas control muscles (**C**) exhibit the normal arrangement of sarcomeres, with well-developed t-tubules (arrowhead) and terminal cisternae (arrows), grade 3 muscles (**D**) exhibit swelling of the longitudinal sarcoplasmic reticulum and the terminal cisternae. Moreover, irregular spacing of adjacent myofibres and serious disruption of some of the myofibres are evident in grade 3 larvae. (**E**,**F**) Retina and brain of representative control (**E**) and grade 3 (**F**) larvae. Grade 3 larvae exhibit liquefactive necrosis at the brain level. (**E’**,**F’**) Higher magnification of the brain at the optic tectum level corresponding to the area indicated by a frame dashed box at (**E**) and (**F**). Whereas the control larva exhibits a normal structure in both neuronal bodies and axons (**E’**), the grade 3 larva exhibits pyknotic nuclei and striking alteration of the axons (loss of integrity, granular aspect, faint colour) (**F’**). (**G**–**J**) Characteristics of neuronal bodies (**G, I**) and axons (**H**,**J**) in TEM sections of the brain of representative control (**G**–**H**) and grade 3 (**I–J**) larvae. Whereas severely altered neuronal bodies (nuclear changes associated with necrotic processes) are evident in (**I**), altered axons, some of which are extremely enlarged, are present in (**J**). (**K**) Heatmap of the neuroactive ligand-receptor interaction pathway shows the strong down-regulation of this pathway found in grade 3 larvae. This result is consistent with the severe disruption of the CNS found at the histological level in this phenotype. Scale bars: (**A**,**B**) 100 μm, (**C**,**D**) 1 μm, (**E**,**F**) 100 μm, (**G**,**H**) 2 μm, (**E’**,**F’**) 20 μm, (**I**,**J**) 2 μm.

Grade 3 was the most prevalent phenotype at high CPO concentrations. The primary macroscopic features of this severe phenotype were altered morphology of the head and complete paralysis. Moreover, most of the larvae exhibited some degree of periocular and/or peritoneal edema, and this clinical sign correlated with the severity of the phenotype (Supplementary Fig. S3). Surprisingly, and in contrast to the observed effects in grade 2 larvae, only mild reductions in the lengths of both the axial slow-twitch muscle fibres and trunk were found in grade 3 larvae (Supplementary Fig. S5). Moreover, when the integrity of slow-twitch muscle fibres was analysed, groups of damaged fibres were found at different locations along the trunk. The observed lesions were consistent with rupture of muscle fibres following violent jerking of the trunk during initial seizures. Histopathological analysis also detected generalized degeneration and necrosis of the fast-twitch muscle fibres (Fig. 6A,B). When the ultrastructure of axial muscle fibres was analysed by transmission electron microscopy (TEM), diffuse cytoplasmic swelling, with dilation of the sarcoplasmic reticulum and degeneration of some myofibers, became evident (Fig. 6C,D). In contrast with the severity of the lesions found in organs receiving cholinergic innervation, such as CNS or muscle fibres, non-cholinergic tissues, such as the liver, remained well preserved (Supplementary Fig. S13).

Protracted seizure activity in severe OPP produces neuronal necrosis that culminates in liquefactive necrosis^4^ in rats. Widespread liquefactive necrosis with disruption of the local architecture was found in the brain and spinal cord of grade 3 larvae (Fig. 6E,F’ and Supplementary Fig. S14). Analysis of the brain at the ultrastructural level showed nuclear changes associated with necrosis in the neuronal bodies and severely altered axons (Fig. 6G–J). Moreover, this grade exhibited the most severe damage in the retina, with retinal detachment and generalized cell death in the granular cell layer and the inner and outer nuclear layers (Fig. 2A,D).

In rats exposed to a single dose of a potent OP compound, such as soman, myotoxic and neurodegenerative effects were already present during the first 3 h, and the severity of the condition increased significantly at 24 h post-exposure^4^. The commitment of larvae exposed to 3 μΜ CPO to present a grade 3 phenotype occurs during the first 3 h of exposure, and then the fate of the animals is irreversible. No differences in the prevalence of the grade 3 phenotype were found between larvae exposed continuously for 24 h and those exposed for 3 h and then transferred to clean water for an additional 21 h (Supplementary Fig. S15).

**Figure 7:**
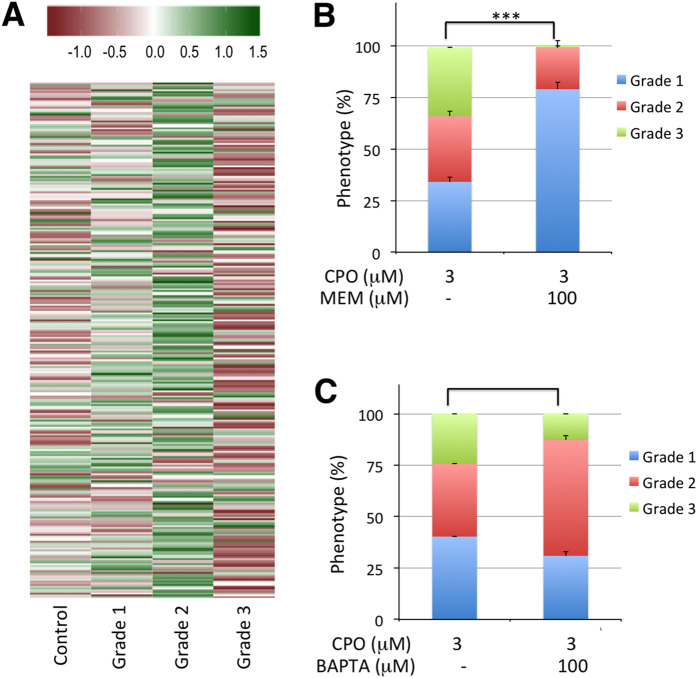
Calcium dysregulation is central to grade 3 development. (**A**) Heatmap of the calcium signalling pathway (dre04020) in control and the different grades of OPP, showing clear down-regulation of this pathway in grade 3 larvae. (**B)** Memantine, an antagonist of NMDA receptors, reduced the prevalence of the grade 3 phenotype dramatically [9 groups with 10–18 larvae (total larvae: 132) were analysed for 3 μΜ CPO and 9 groups with 13–25 larvae (total larvae: 200) for 3 μΜ CPO + 100 μΜ memantine; p < 0.001, Mann-Whitney U Statistic (T(9) = 126)]. Seven days post fertilization (dpf) larvae were pre-incubated with 100 μM memantine for 1 h followed by co-exposure with 100 μM memantine/3 μM CPO for 24 h. (**C**) BAPTA-AM, a permeable calcium chelator, also reduced the prevalence of the grade 3 phenotype significantly [7 groups with 12–42 larvae each (total larvae: 214) were analysed for 3 μΜ CPO and 5 groups with 11–18 larvae (total larvae: 70) for 3 μΜ CPO + 100 μΜ BAPTA-AM; p < 0.005, Student’s *t*-test (t(12) = 3.287)]. Six days post fertilization zebrafish larvae were pre-incubated with 100 μM BAPTA-AM for 24 h followed by 24 h co-exposure with 3 μM CPO.

RNA-seq analysis of grade 3 larvae identified 4,996 DEGs (Supplementary Data 6). Only nine pathways were significantly down-regulated (FDR-adjusted p ≤ 0.05; Supplementary Data 7). First, the strong down-regulation found in the neuroactive ligand-receptor interaction pathway (dre04080) could be explained, at least partially, by the massive cell death observed at the CNS level. Moreover, the strong down-regulation found in phototransduction-related genes (Fig. 2E), including the opsin 1 family (Supplementary Data 6), is consistent with cell death and strong disruption of the retina architecture in these animals. The strong down-regulation of the calcium signalling pathway (dre04020; Fig. 7A), including most of the genes involved in Ca^2+^ homeostasis, suggests a critical decrease in the Ca^2+^ buffer capacity in grade 3 larvae. Thirty-four pathways were significantly up-regulated in grade 3 larvae (Supplementary Data 8), 28 of which were common with grade 2 larvae. Moreover, similar to the moderate phenotype, the up-regulated pathways included most of those pathways involved in inflammatory and immune responses.

Once the severe phenotype was characterized, we decided to further analyse the pathophysiological mechanisms involved in the generation of this form of OPP. The presence of necrosis in both the CNS and axial muscle fibres, together with the transcriptomic profile, suggests the uncontrolled activation of calcium-activated proteases, phospholipases and kinases by the fatal combination of a massive influx of extracellular Ca^2+^ through activated NMDA receptors and the compromised Ca^2+^ buffer capacity. The first key event in this suggested mechanism, upstream of NMDA activation, is AChE inhibition and AChR overstimulation. To test this hypothesis, larvae were co-exposed to 3 μΜ CPO and 200 μM 2-PAM, and a partial recovery of AChE activity with a full rescue of the grade 3 phenotype was found (Supplementary Fig. S11). The second key event in this hypothesis, immediately after cholinergic seizures, is activation of NMDA receptors. When larvae were pre-treated for 1 h with 100 μΜ memantine, an NMDA receptor antagonist, and later co-exposed to 3 μΜ CPO/100 μΜ memantine for an additional 24 h, the prevalence of the grade 3 phenotype decreased from 33.4 ± 3.3% to 0.93 ± 0.93% (Fig. 7B). Moreover, memantine reduced the 89% mortality induced by 3 μΜ CPO. The almost total recovery found after blocking the NMDA receptors clearly demonstrate that these receptors play a central role in grade 3 pathophysiology. NMDA receptors are calcium channels, and overstimulation of these receptors should induce an excessive accumulation of intracellular Ca^2+^ and activation of different calcium-dependent enzymes, resulting in the observed damage at cellular and subcellular levels. When larvae were pre-exposed (6–7 dpf) to 100 μΜ BAPTA-AM, a permeable calcium chelator, for 24 h and later co-exposed to 3 μΜ CPO and 100 μΜ BAPTA-AM, a 48% decrease in the prevalence of the grade 3 phenotype was found (Fig. 7C). These data support the hypothesis that after initial cholinergic overstimulation, an influx of extracellular Ca^2+^ through NMDA receptors occurs and that the increase in the cytoplasmic levels of Ca^2+^ in animals with a compromised Ca^2+^ buffer capacity results in the uncontrolled activation of proteases, phospholipases and kinases.

Once the role of calcium dysregulation in the development of the severe OPP phenotype in zebrafish was determined, we then explored the potential presence of oxidative stress. When the levels of endogenous GSH, SOD and catalase activities were measured, we found that all these antioxidant systems were significantly altered in grade 3 larvae (Fig. 4A). Dysregulation of the antioxidant defence system was significantly more potent in grade 3 larvae than in grade 1 or 2 larvae (Fig. 4A). Importantly, in grade 3 larvae, the antioxidant defence system was altered, and ROS generation was detected in live larvae using the fluorescent dye DCFH-DA (Fig. 4B–G). ROS generation was found in tissues receiving cholinergic innervation, such as axial muscle fibres and the spinal cord, but not in other tissues without this cholinergic input, such as notochord. Finally, when the presence of oxidative stress was evaluated by measuring lipid peroxidation, grade 3 larvae exhibited increased levels of oxidative stress with respect to the control larvae (Fig. 4A).

Once the presence of oxidative stress was determined in our model for severe OPP, the potential source of ROS was explored. The mitochondrion is an important sub-cellular target of OPs as a non-cholinergic mechanism of toxicity. Mitochondrial complex I inhibition by toxicants can directly result in increased oxidative stress, particularly through ROS production, in addition to reduced mitochondrial function^29^. An increase in ROS production concomitant with inhibition of mitochondrial complex I activity has also been found after exposure to chlorpyrifos both *in vitro* and *in vivo*^30^. Thus, we decided to test the potential disruption of the mitochondrial function in grade 3 larvae. First, swelling and loss of cristae in the mitochondria from the CNS and axial muscle of grade 3 larvae were observed by transmission electron microscopy (Supplementary Figs S16, S17). Moreover, analysis of mitochondrial respiration (Fig. 4H–J) showed strong reductions in basal, maximal and coupled respiration in grade 3 larvae. Considering that complex I substrates were used for the basal respiration analysis, the strong reductions found in grade 3 larvae reflected the dysfunction of complexes I, III and/or IV, which are major sites for ROS production^31^. Therefore, CPO exposure-induced dysfunction in mitochondrial respiration in grade 3 larvae was the most suitable explanation for the observed increase in ROS generation in this phenotype.

Our next biological question was to understand if oxidative stress plays an essential role as a non-cholinergic mechanism in the development of severe OPP. If this hypothesis was true, then protecting larvae with antioxidants should at least partially rescue the phenotype. Therefore, we analysed the effect of modulating the endogenous GSH levels on the prevalence of the grade 3 phenotype. N-acetylcysteine (NAC) was selected as the antioxidant and glutathione as the pro-drug, whereas diethyl maleate (DEM) was selected to decrease the endogenous GSH levels. We found that pre-incubation of the larvae for 24 h (6–7 dpf) with 50 μΜ NAC induced a 9% increase in the endogenous GSH levels and that when these larvae were exposed to 3 μΜ CPO, the prevalence of grade 3 phenotype decreased by 67% (Fig. 4K,L). Moreover, pre-treatment with 0.5 μΜ DEM induced a 12% decrease in the endogenous GSH levels, and when these larvae were exposed to 3 μΜ CPO, the prevalence of the grade 3 phenotype increased by 82% (Supplementary Fig. S18). Thus, these results demonstrated that endogenous GSH levels were able to modulate the prevalence of the grade 3 phenotype. Nevertheless, GSH is not only one of the most important antioxidants but also involved in numerous processes that are essential for normal biological functions, including xenobiotic detoxification^2^. To clarify the role of oxidative stress in the development of the grade 3 phenotype, the effects of the mitochondria-targeted antioxidant MitoQ, the natural antioxidant ascorbic acid (vitamin C) and a water-soluble vitamin E analogue (Trolox) were assessed. Unexpectedly, none of these three antioxidants decreased the prevalence of the grade 3 phenotype significantly (p > 0.05, Student’s *t*-test) (Fig. 4M,N and Supplementary Fig. S19). These results strongly suggested that although oxidative stress is produced during the development of the grade 3 phenotype, this process is not essential to the pathophysiology of this grade of OPP. Moreover, our data indicated that the effect of GSH on the recovery of the grade 3 phenotype was mediated through an oxidative stress-independent mechanism.

Development and validation of animal models for rapid screening of molecular libraries to identify potential medical countermeasures against OPP are some of the primary priorities of NATO countries^11^. Zebrafish is a vertebrate suitable for use as an animal model for human diseases in high-throughput screening of small molecule libraries. Nevertheless, before using any zebrafish model of human OPP, the validity of the developed model must be analysed^19^. To test the validity of a proposed zebrafish model for OPP, the clinical features of this toxidrome in the model must be characterized, and the pathophysiological pathways leading to the observed clinical features must be analysed. The zebrafish models for OPP described here recapitulate the most relevant pathophysiological mechanisms described in humans, representing a powerful tool for the discovery of new drugs to improve the survival rate and the clinical condition of patients with OPP. We have shown that CPO is a potent inhibitor of AChE activity in zebrafish larvae and that this inhibitory action is an essential step in the pathophysiology of the different OPP grades. Furthermore, the results of the involvement of NMDA receptors, along the transcriptomic data regarding dysregulation of calcium and activation of inflammatory and immune responses in our model of severe OPP, support common mechanisms of toxicity of OP compounds between mammals and zebrafish (Supplementary Discussion). The animal models developed in this study could be classified as induced models of OPP, and the analysis of the clinical features, adverse effects and toxic pathways demonstrated that these models could be classified as partial models, defined as models that do not mimic the entire human disease but that may be used to study certain aspects or treatments of the disease^19^. Therefore, our study suggests that the developed zebrafish models of OPP can be used for the identification of new antidotes or combinations of antidotes against this toxidrome.

## Methods

### Zebrafish stocks and larvae production

Embryos from wild-type zebrafish (Supplementary Methods) were obtained by natural mating and maintained in fish water at 28.5 °C. Larvae were maintained under starvation conditions during the entire experimental period, including recovery periods. All procedures were conducted in accordance with institutional guidelines under a license from the local government (DAMM 7669, 7964) and approved by the Institutional Animal Care and Use Committees at the Research and Development Centre of the Spanish Research Council (CID-CSIC) and University of Bordeaux.

### Stability of CPO in water determination

The stability of CPO in fish water under exposure conditions was tested by LC-MS/MS (see Supplementary Methods for additional details).

### Biochemical determination

AChE activity was determined in individuals, while SOD, CAT and GSH were determined in pools of either 5 or 10 larvae. Larvae were homogenized by ultrasound in ice-cold 0.1 M phosphate buffer (pH 7.4) with 150 mM KCl and 0.1 mM EDTA and further centrifuged at 10,000 × g for 10 min at 4 °C. The resulting supernatant was collected for AChE, SOD, CAT and GSH analysis. All biochemical measurements were conducted at 25 °C using a microplate reader (Supplementary Methods).

### LPO determination

LPO was determined by quantifying the levels of malondialdehyde following standard protocols (Supplementary Methods).

### *In vivo* detection of ROS generation

A 8 mM stock of 2′,7′–dichlorofluorescein diacetate (DCFH-DA, Sigma) was prepared in DMSO, aliquoted and stored at −80 °C. For *in vivo* ROS detection, control and grade 2 and 3 larvae were incubated in 20 μM DCFH-DA (0.5% DMSO in the incubation medium) for 1 hour in the dark at 28.5 °C. After the larvae were washed 5 times for 5 min with fish water, fluorescence was observed under 488 nm excitation using a Nikon Eclipse 90i microscope fitted with a Nikon Intensilight C-HGFI unit.

### Histopathological evaluation

Standard protocols were used for the histopathological analysis by light microscopy and transmission electron microscopy (Supplementary Methods).

### Behaviour

Basal locomotor activity and the VMR of 8 dpf zebrafish larvae were analysed using a DanioVision system running EthoVision XT 9 software (Noldus, Wageningen, the Netherlands) essentially as described elsewhere (see Supplementary Methods for additional details). For TMR, startle responses were recorded with a high-speed Photron Fastcam SA3 camera (Photron USA, Inc., San Diego, CA, USA), and the kinematics of the response was analysed using the Flote software package (Supplementary Methods).

## Molecular Biology

### Illumina TruSeq RNA Library Preparation

Total RNA was isolated from either individual larvae or pools of 5 to 8 larvae after thorough homogenization using a NucleoSpin RNA XS kit (Macherey-Nagel, GmbH & Co. KG) following the manufacturer’s recommendations. Library preparation and sequencing were performed by Global Biologics LLC (Columbia, MO, USA). Total RNA was quantitated using a Qubit RNA assay kit and Qubit 2.0 fluorometer (Life Technologies Inc.), and RNA integrity was confirmed using the standard sensitivity Fragment Analyzer Total RNA Assay and System (Advanced Analytical Inc.). Briefly, five hundred nanograms of total RNA was used as input material for the Illumina TruSeq Directional v2 high-throughput library construction procedure (Illumina Inc.). Messenger RNA was enriched from total RNA using oligo-dT magnetic beads and fragmented to ~100–300 bp with a single shearing and RT primer hybridization step before generating first- and second-strand cDNA. The resulting DNA was prepared for sequencing by blunt end repair, 3′ adenylation, multiplex compatible adapter ligation (containing TruSeq indexes), and PCR amplification (98 °C for 30 sec, 11–13 cycles [98 °C for 10 sec, 60 °C for 30 sec, and 72 °C for 30 sec], 72 °C for 5 min, and 10 °C hold). Library validation was performed using the Fragment Analyzer (Advanced Analytical Inc.) followed by quantitation using the Qubit HS DNA Assay and qPCR Kit for Illumina (Kapa Biosystems Inc). Libraries were diluted using the Qubit or qPCR information and sequenced using four lanes (single-read 100 bp sequencing) on the HiSeq 2500 platform (Illumina Inc).

### RNA-seq

Sixteen samples (4 biological replicates for 4 treatments) were mapped to the *Danio rerio* genome GRCz10 from NCBI using Tophat v2.0.13^32^. The number of uniquely mapped reads for each library can be found in Supplementary Data 1. Any read with more than two mismatches in the best alignment or that aligned to more than 20 regions in the genome were discarded. Estimated gene expression levels were produced by htseq v0.6.1p1^33^ from the accepted hits from Tophat. Genes with very low expression (less than 2 counts per million reads in more than 4 replicates) were removed from further analysis^34^. In total, 29,976 annotations passed filtering. The gene expression estimates were then normalized based on the sequencing depth of each library. EdgeR v3.2.4^35^ was used to find differentially expressed genes. Generalized linear models were used to test for differential expression based on comparing each treatment against the control. Genes with a FDR-adjusted p-value ≤ 0.05 were considered differentially expressed. Gage v2.14.4^36^ was used to find significantly perturbed KEGG pathways. Each comparison was tested for significant pathways using the above-estimated gene expression levels. Any pathway with a FDR-adjusted p-value ≤ 0.05 was considered significant. These sequencing data are archived in the NCBI Short Read Archive with the BioProject accession number PRJNA285816.

### Measurement of oxygen consumption in zebrafish larvae homogenates

Respiration of zebrafish homogenates (400–500 μg protein) was measured at 28 °C by high-resolution respirometry (Supplementary Methods).

### Measurement of the adenine nucleotide levels

AMP, ADP and ATP levels were determined by reverse-phase HPLC using a C18 column (Supplementary Methods).

### Concentration-response analysis

EC50 and LC50 values were obtained by fitting responses relative to control treatments (*R*) to the nonlinear allosteric decay regression model (see Supplementary Methods for additional details).

### Data analysis

Statistical analysis was performed using SPSS software v22 (IBM, USA). The data are presented as the mean ± SEM unless stated otherwise. Pairwise statistical significance was determined with Student’s *t*-test, one-way ANOVA or Mann-Whitney rank sum test as appropriate. The results were considered significant at p < 0.05 unless otherwise indicated.

## Additional Information

**How to cite this article**: Faria, M. *et al.* Zebrafish Models for Human Acute Organophosphorus Poisoning. *Sci. Rep.* **5**, 15591; doi: 10.1038/srep15591 (2015).

## Supplementary Material

Supplementary Information

Supplementary Movie 1

Supplementary Movie 2

Supplementary Dataset 1

Supplementary Dataset 2

Supplementary Dataset 3

Supplementary Dataset 4

Supplementary Dataset 5

Supplementary Dataset 6

Supplementary Dataset 7

Supplementary Dataset 8

Supplementary Dataset 9
