# Corrigendum: Zebrafish Models for Human Acute Organophosphorus Poisoning

**DOI:** 10.1038/srep17244

**Published:** 2016-01-07

**Authors:** Melissa Faria, Natàlia Garcia-Reyero, Francesc Padrós, Patrick J. Babin, David Sebastián, Jérôme Cachot, Eva Prats, Mark Arick, Eduardo Rial, Anja Knoll-Gellida, Guilaine Mathieu, Florane Le Bihanic, B. Lynn Escalon, Antonio Zorzano, Amadeu M. V. M Soares, Demetrio Raldúa

Scientific Reports 5: Article number: 1559110.1038/srep15591; published online: 10222015; updated: 01072016.

In this Article, Mark Arick II and B. Lynn Escalon are incorrectly listed as being affiliated with ‘Environmental Laboratory, US Army Engineer Research and Development Center, Vicksburg, MS, USA’ and ‘EPOC, UMR CNRS 5805, Université de Bordeaux, 33405 Talence, France’ respectively. The correct affiliations are listed below:

Mark Arick II

Institute for Genomics, Biocomputing & Biotechnology (IGBB), Mississippi State University, Starkville, Mississippi, USA

B. Lynn Escalon

Environmental Laboratory, US Army Engineer Research and Development Center, Vicksburg, MS, USA

