# Supplementary material for: Zebrafish Models for Human Acute Organophosphorus Poisoning
Source: Sci Rep. 2015 Oct 22;5:15591. doi: 10.1038/srep15591 (PMC4614985; doi:10.1038/srep15591)
Supplement: Supplementary Information [file srep15591-s1.pdf]

SUPPLEMENTARY INFORMATION

Zebrafish Models For Human Acute Organophosphorus Poisoning

Melissa Faria, Natàlia Garcia-Reyero, Francesc Padrós, Patrick J. Babin, David Sebastián, Jérôme Cachot, Eva Prats, Mark Arick II, Eduardo Rial, Anja Knoll-Gellida, Guilaine Mathieu, Florane Le Bihanic, B. Lynn Escalon, Antonio Zorzano, Amadeu M.V.M Soares, Demetrio Raldúa[1]

[1]To whom correspondence should be addressed. Email: drpqam@cid.csic.es

This file includes:

SI Method ……………………………………………..2-13

SI Discussion ……………………………………14-17

SI Figures – Figures S1 to S17 …………….. 18-37

SI Tables – Tables S1 and S2 ………………..38-39

SI Movie Legends …………………………..……40-40

SI References ……………………..………………. 41-43

Supplementary Methods

Zebrafish stocks and larvae production

Wild-type zebrafish were obtained from Piscicultura Superior (Barcelona, Spain) and maintained in fish water [reverse-osmosis purified water containing 90 µg/mL of Instant Ocean (Aquarium Systems, Sarrebourg, France), 0.58 mM $CaSO_4 \cdot 2H_2O$] at 28±1°C in our facilities under standard conditions.

Stability of CPO in water determination

Nominal working solutions of 0.1 (33.3 ppb), 1 (333 ppb) and 3 µM (1000 ppb) were prepared in fish water as described above with n=3 replicates per treatment. Working solutions were incubated with larvae under identical exposure conditions. Water samples were collected at time 0 and 24 hours of exposure and immediately analysed by LC-MS/MS (TqDetector, Acquity Waters, USA). Pure analytical standards of 98–99% purity of methanol (MeOH), acetonitrile (ACN), and HPLC water (LiChrosolv grade) were supplied by Merck (Darmstadt, Germany). CPO standard was prepared in fish water following exhaustive control of handling procedures, storage conditions and safety rules. The analyses were performed using a Luna C18 (150 mm×2 mm ID, particle size 5 µm, Phenomenex, Torrance, USA) equipped with a Security Guard pre-column. The mobile phase composition consisted of binary mixtures with 0.1% formic acid in ACN (A) and 0.1% formic acid in water (B). The system was operated at room temperature, the flow rate was set at 200 µL min^{-1}, and samples were analysed under positive electrospray ionization mode (ESI+). The gradient profile was 0-4 min (A, 5 to 40%; B, 95 to 60%), 4-7 min (A, 40 to 60%; B, 60 to 40%), 7-11 min (A, 60 to

100%; B, 40 to 0%), 11-15 min (A, 5 to 100%; B, 0 to 95%). The flow rate was 200 μL/min, and the injection volume was 10 μL. Acquisition was performed in SRM mode using three transitions from [M-H]+ precursor ion to daughter ions to identify the compound. The data were acquired and processed using the MassLynx v4.1 software package.

OPP model generation

A 150 mM stock of CPO (ChemService, UK) was prepared in DMSO, aliquoted and stored at -20°C. For OPP model generation, starved 7 dpf zebrafish larvae were transferred to 48-well plates (1 larva per well) and exposed to selected CPO concentrations in a dark incubator at 28.5°C for 24 h. Unless stated otherwise, grade 1, 2 and 3 larvae were generated with 0.1, 1.0 and 3.0 μM CPO, respectively. Control larvae were exposed under identical conditions to the same concentration of the carrier (0.1% DMSO).

Morphometric analyses

Phenotypes were recorded as exhibited by each animal at the indicated CPO concentration and exposure time. Live larvae were examined with a Nikon SMZ 1500 stereomicroscope to observe their phenotypes. Images and video were acquired with a Nikon Digital Sight DS-Ri1 camera and NIS Elements AR software (version 3.0), and images were saved as high-resolution (3840 pixels x 3005 pixels) tagged image file format (TIFF). Images of the slow muscle fibres stained with F59 antibody were acquired with a Nikon Eclipse 90i microscope fitted with a Nikon Intensilight C-HGFI unit. All images for morphometric analysis had a constant number of pixels per inch. Trunk length, the distance between the tip of the tail and the most caudal part of the swim bladder, and slow muscle fibre length

were estimated using the free image analysis software ImageJ (U. S. National Institutes of Health, Bethesda, Maryland, USA, http://imagej.nih.gov/ij/), as indicated in SI Fig. S5 *A* and *B.*

Biochemical determination

AChE activity was determined by adding 2 mM acetylthiocholine and 0.33 mM 5,5'-dithiobis-(2-nitrobenzoic acid) (DTNB) to the S9 fraction and monitoring the formation of the product resulting from the reaction between thiocholine and DTNB ion at 405 nm for 15 min. The final results were expressed in μmol min^{-1} mg protein using the extinction coefficient 13.6 × 10^3 M^{-1} cm^{-1}. SOD activity assessment was based on the measurement of the degree of SOD-induced inhibition of the reduction of cytochrome c by free oxygen radicals (O^{2-}) released by the xanthine oxidase/xanthine reaction. SOD units were determined using a standard curve of 0-1.5 SOD units/mL. Reactions of sample and standard measured in quadruplicate occurred under identical conditions. Cytochrome c (0.01 mM) and xanthine (0.023 mM, which were both prepared in 50 mM phosphate buffer (pH 7.8) and 0.1 mM EDTA, were added to each sample and standard. The reaction was initiated by adding xanthine oxidase (0.0017 U mL^{-1}) and measured for 5 min at 550 nm at 25°C. The final results were normalized by the total protein content and expressed as U mg^{-1} of total protein. CAT activity was measured by the decrease in absorbance at 240 nm due to H_2O_2 consumption (extinction coefficient, 40 M^{-1} cm^{-1}). Total glutathione (GSH) quantification was measured by incubating samples for 60 min at room temperature with 0.1 mM monochlorobimane (mCB) and 1 U mL^{-1} glutathione *S*-transferase (GST). The reduced glutathione present in the cells forms a GSH-mCB complex aided by the

GST enzyme. The resulting complex is then measured fluorometrically at an excitation:emission wavelength of 360 nm:460 nm. The reduced GSH content was then extrapolated from a GSH standard curve determined under the same conditions as the samples. The final results were expressed as nmol mg of protein^{-1}.

LPO determination

LPO was determined by quantifying the levels of malondialdehyde (MDA). The MDA assay is based on the reaction of the chromogenic reagent 1-methyl- 2-phenylindole with MDA at 45°C, giving rise to a chromophore with absorbance at 586 nm. Pools of 50 to 70 individuals were homogenized in ice-cold 0.1 M phosphate buffer (pH 7.4) containing 0.01% 2,6-di-tert-butyl-4-methylphenol (BHT). Homogenates were incubated with 5 mM 1-methyl-2-phenylindole in acetonitrile:methanol (3:1 v/v), 5.55% HCl and 0.01% BHT at 45°C, for 45 min. Absorbance was read at 586 nm, and MDA content in each sample was extrapolated from the standard curve of 1,1,3,3-tetramethoxypropane (TMP) treated under similar conditions as samples. The final results were normalized by mg of total lipid contents for each sample and expressed as nmol mg protein^{-1}. Total protein was measured by the Bradford method using bovine serum albumin (BSA) as the standard.

Histopathological evaluation

Light Microscopy. Larvae were fixed in 10% phosphate-buffered formalin (pH 7.2) at room temperature. Subsequently, the larvae were dehydrated in a graded ethanol series (30, 50, 70, 90, 96 and 100%) for 30 min each. The larvae were progressively embedded in infiltration solution (Technovit® 7100, Kulzer) using

absolute ethanol (1:3, 1:1 and 3:1) for 1 h in each step. Then, the larvae were placed in 100% infiltration solution overnight at room temperature. The specimens were placed in Teflon block moulding cups (Kulzer), and the remaining infiltration solution was removed using a pipette. New infiltration solution with hardener II (15:1) was added to the cups and placed in an incubator (37°C). After polymerization, hardened methacrylate blocks were glued to plastic holders using Technovit® 3040 (Kulzer) and removed from the cups. Blocks were sectioned with tungsten knives in a rotary microtome (Reichert-Jung). Sections (2 µm thick) were floated on a distilled water bath at room temperature, picked up on clean glass slides and dried at 40°C. Sections were immersed in an aqueous solution of 1% Toluidine Blue for 2 min and rinsed in tap water until the desired blue intensity and contrast with the background. The sections were dried at 37°C and mounted using DPX.

Transmission electron microscopy. Whole control and P3 larvae were fixed in 2.5% glutaraldehyde in 0.1 M cacodylate buffer (pH 7.4) at 4°C and postfixed in 1% osmium tetroxide. The larvae were embedded in Eponate 12™ resin (Ted Pella Inc.) and polymerized at 60°C for 48 h. Semi-thin sections (1 µm) were obtained with a Leica Ultracut UCT Microtome (Leica Microsystems GmbH). Ultra-thin sections (70 nm) were mounted on copper grids and stained with uranyl acetate (30 min) and Reynolds' lead citrate (5 min) solutions. Sections were observed with Hitachi H-7000 and Jeol-1400 transmission electron microscopes equipped with Gatan Erlangshen CCD cameras.

Pharmacological treatments

The following drugs, which were obtained from Sigma-Aldrich, were used in the

pharmacological approach: pralidoxime (2-PAM), N-acetylcysteine (NAC), diethyl maleate (DEM), ascorbic acid, 6-hydroxy-2,5,7,8-tetramethylchroman-2-carboxylic acid (trolox), memantine, and 1,2-Bis(2-aminophenoxy)ethane-N,N,N′,N′-tetraacetic acid tetrakis(acetoxymethyl ester) (BAPTA-AM). MitoQ was a kind gift from Dr. Mike Murphy (University of Cambridge, UK). No toxicity was found for all compounds used at the indicated working concentrations. Stock solutions of 2-PAM, NAC, DEM, trolox and BAPTA-AM were prepared in DMSO and then diluted to test concentrations in fish water. Solutions of ascorbic acid, MitoQ and memantine were prepared directly in fish water. The final concentrations of the carrier (DMSO) in the incubation media ranged from 0.1 to 1%.

First, the effect of 2-PAM on AChE activity and on the prevalence of the different phenotypes was analysed by conducting co-exposure experiments with different concentrations of CPO (0.1, 1, 3 and 6 μM) and 2-PAM (1 to 3200 μM) for 24 h. No pre-incubation step was included. The concentration of the carrier (DMSO) was 0.1% for all the assayed conditions. Following exposure, the survival and prevalence of the different phenotypes were recorded, and AChE activity was analysed.

The role of GSH in the development of grade 2 and 3 phenotypes was explored by conducting co-exposure experiments with CPO (1 and 3 μM) and NAC (25, 50 and 100 μM) or DEM (0.05, 0.5 and 5 μM). In both cases, larvae were pre-incubated for 24 h in fish water with either NAC or DEM alone, after which some samples of larvae were collected to determine GSH content before co-exposure with CPO. Throughout the pre-incubation and co-exposure, the DMSO concentration was 0.1%. Following the co-exposure, the survival and prevalence of the different

phenotypes were recorded.

The role of oxidative stress in the development of the grade 2 and 3 phenotypes was studied by conducting combined exposures of CPO (1 and 3 μM) with ascorbic acid (vitamin C; 100 and 250 μM), Trolox (vitamin E water-soluble analogue; 50 and 100 μM) and MitoQ, a mitochondria-targeted antioxidant (1 μM). Before the addition of CPO, the larvae were first pre-incubated in fish water with antioxidants for 3 hours (ascorbic acid and trolox) or 24 hours (MitoQ). The DMSO concentration during pre-incubation/co-exposure was 0/0.1%, 0.5/0.1% and 1/0.1% for ascorbic acid, trolox and MitoQ, respectively.

The roles of NMDA receptors and intracellular calcium in the development of the grade 3 phenotype were examined using memantine hydrochloride, a NMDA receptor antagonist, and BAPTA-AM, a permeable calcium chelator. Zebrafish larvae were placed in fish water with either CPO (1 and 3 μM) alone or together with memantine (100 μM) or BAPTA-AM (100 or 200 μM). In both cases, the larvae were pre-incubated in fish water containing only memantine (100 μM) or BAPTA-AM (100 and 200 μM) for 1 hour and 24 hours, respectively. After co-exposure, the survival and prevalence of the different grades of OPP were recorded for all treatments.

Behaviour analysis

Basal locomotor activity and VMR of 8 dpf zebrafish larvae were analysed essentially as described elsewhere[1]. At the end of the 24 h exposure period, 8 to 16 larvae from each exposure or control condition were placed in 48- well microplates (Nunc™) containing 1 mL of experimental solution per well (internal diameter of 12 mm, flat bottom). The plate was then transferred into a behavioural

testing chamber equipped with a temperature control unit (DanioVision, Noldus Information Technology, Leesburg, VA). Larvae were acclimated in the dark for 1 h before video recording. The video tracking conditions used were a 50 min cycle including a 20 min dark period followed by a 10 min light period and then a second cycle of 20 min of darkness. The position of each individual larva was then recorded using an IR digital video camera (Ikegami Electronics, Neuss, Germany) and an EthoVision XT 9 video tracking system (Noldus Information Technology, Leesburg, VA). A dynamic subtraction method was applied, using a sampling rate of 60 images/s, dark contrast 20-250, current frame weight 1, subject-size 2-125000, and no subject contour dilatation. A minimum distance input with a filter of 10% of the total larva body, equivalent to 0.4 mm, was used to remove background noise. Larvae with any gross morphology defect or dead larvae were not included in this analysis. All measurements occurred in the afternoon between 1:00 and 6:00 pm, the optimal time interval for the stability of the basal activity. Tracks were analysed for velocity (mm s-1), total distance moved (m) and mobility time (s) calculated for each dark or light period. Mobility time refers to the period where 20 to 60% of the total body of the larva is modified even if the central point does not change. All microplates were analysed at 20 ± 0.5°C with same detection and acquisition settings.

For TMR, startle responses were evoked in 8 dpf larvae by a light-touch stimulus applied to the rostral head skin using a glass capillary injection needle. Video recordings were initiated 2 min after moving the larva to the testing arena to allow sufficient time for locomotor activity to stabilize. All video recordings were made with a high-speed Photron Fastcam SA3 camera (Photron USA Inc., San Diego, CA, USA) at 512 × 512 pixel resolution using a Sigma 105 mm F2.8 EX DG lens at 1000

frames per second. Individual larvae were tested in 6 cm Petri dishes. The plate was illuminated from below by a LLUB White LED Backlight 50×50 (PHLOX, Aix-en-Provence, France) adjusted to 300 μW/cm^2 using a Gardasoft RT 220-20 LED Lighting Controller (Gardasoft, Cambridge, UK). The light intensity in the testing platform was measured using an ILT1400 radiometer (International Light Technologies Inc., Peabody, MA, USA). All behavioural measurements were made using the Flote software package. Briefly, this software performs tracking of the filmed larva and then performs an automated analysis of its body curvature to extract kinematic details of swimming movements. The curvature of the body was calculated for each frame and plotted over time using Microsoft Excel (Microsoft Corporation, Redmon, WA, USA).

Whole-mount immunofluorescence

Larvae were fixed in 4% PFA in PBS overnight at 4°C, dehydrated through a methanol gradient and stored in absolute methanol at -20°C. After rehydration, the larvae were depigmented (3% H_2O_2 and 1% KOH in water, 35 min) and permeabilized by immersion in -20°C acetone (12 min) and treatment with 0.1% collagenase (Sigma, C-9891) in PBS (pH 7.4) at room temperature for 20 min. Then, the larvae were pre-incubated in blocking buffer (4% goat serum, 1% BSA, 1% DMSO, 0.8% Triton X-100, and 0.1% Tween-20 in PBS, pH 7.4) for 2 h and incubated overnight at 4°C in a mixture of mouse monoclonal anti-acetylated tubulin antibody (IgG2b; Sigma) at 1:1000 and mouse monoclonal F59 antibody (IgG1; DSHB, University of Iowa, Iowa) at 1:50. These antibodies were used to detect spinal axonal tracts and slow muscle fibres, respectively. Then, the larvae were washed for 3 h and incubated for 2 h in a mixture of secondary antibodies

(Alexa 488-conjugated goat anti-mouse IgG2b and Alexa 594-conjugated goat anti-mouse IgG1), at 1:300. After the larvae were washed for 3 h, they were imaged on a Nikon Eclipse 90i microscope fitted with a Nikon Intensilight C-HGFI unit.

Measurement of oxygen consumption in zebrafish larvae homogenates

Respiration of zebrafish homogenates (400-500 µg protein) was measured at 28°C by high-resolution respirometry using an Oxygraph-2k (OROBOROS INSTRUMENTS, Innsbruck, Austria). Homogenates were obtained by sonicating 20-25 larvae in buffer II (0.5 M sucrose, 50 mM KCl, 5 mM EDTA, 1 mM sodium pyrophosphate, 5 mM $MgCl_2$ [pH 7.4], and protease inhibitors) and centrifuged at 1,000 × g for 10 min at 4°C. In total, 500 µl of the supernatant, the mitochondrial enriched extract, was brought to a final volume of 2.1 mL with respiration medium (0.5 mM EGTA, 3 mM $MgCl_2 \cdot 6H_2O$, 20 mM taurine, 10 mM KH_2P0_4, 20 mM HEPES, BSA 1 g/l, 60 mM K-lactobionate, and 110 mM sucrose, pH 7.1) and added to the oxygraph chamber. All respiration measurements were performed in triplicate. Basal respiration (state 3) was assessed by the addition of 10 mM glutamate, 2 mM malate as the complex I substrate supply and 2.5 mM ADP. Respiration uncoupled to ATP synthesis (leak) was assessed by adding 2 µg/mL of the complex V inhibitor oligomycin. Maximal respiration was determined through the addition of the protonophore carbonylcyanide-4-(trifluoromethoxy)-phenylhydrazone (FCCP) (optimum concentration for maximal flux). The addition of 0.5 µM rotenone and 2.5 µM antimycin A resulted in the inhibition of complex I and complex III to observe non-mitochondrial respiration. The concentrations of substrates and inhibitors used were based on prior experiments conducted for optimization of the titration protocols.

Measurement of the adenine nucleotide levels

AMP, ADP and ATP levels were determined by reverse-phase HPLC using a C18 column (Mediterranea SEA 18, Teknokroma, Sant Cugat, Spain). Pools of 6 larvae were washed in fish water and homogenized in 660 mM $HClO_4$ and 10 mM theophylline. Homogenates were centrifuged for 15 min at 16,000×*g*, and then supernatants were neutralized using 2.8 M K_3PO_4, spun to remove the precipitates, filtered and analysed using a Shimadzu Prominence chromatograph (Canby, OR). Peak identities were confirmed by comparison with the retention times of standard adenine nucleotides. Extracts from larvae exposed to 5 μM oligomycin plus 40 mM 2-DOG were used to validate the protocol (see Supplementary Figure S10).

Concentration-response analysis

EC50 and LC50 values were obtained by fitting responses relative to control treatments (*R*) to the nonlinear allosteric decay regression model depicted in equation 1:

$$R(C_i) = \frac{1}{1 + \frac{C_i^k}{\mathrm{EC/LC50}^k}}$$

where

$R(C_i)$ proportional biological response at concentration C_i relative to controls

C_i concentration of the toxic substance (i)

EC/LC50 the half saturation constant (EC is the concentration of CPO that caused an inhibition of 50% of AChE activity, and LC is the

	concentration of CPO that caused 50% mortality of either larvae or adult zebrafish)
k	decay index

The allosteric decay model was selected to fit the obtained data because this model can describe nonlinear type responses. Model accuracy was assessed using the adjusted coefficient of determination (r^2) and analysing residual distribution. The significance of the entire regression and regression coefficients were determined by analysis of variance (ANOVA) and Student's *t*-tests, respectively.

All analyses were conducted using statistical analysis software (IBM SPSS 19.0 and SigmaPlot 11.0, 2008, Systat Software Inc.).

Supplementary Discussion

One of the problems to overcome when animals models are developed to increase our current understanding of the pathophysiological mechanisms involved in OPP is that when animals are exposed to high concentrations of OPs, death occurs very quickly, preventing the expression of any specific phenotype. Most of these early deaths in acute OPP result from acute respiratory failure[2]. Thus, to prevent death, animals are usually pre-treated with a combination of antidotes (atropine and oximes), thus introducing an important confounding factor in the interpretation of the results when the models are used for toxicodynamic studies. Respiratory gas exchange in adult fish occurs by diffusion over the gills[3], and exposure to OPs has been reported to severely decrease oxygen uptake by the gills[4]. The higher resistance of our OPP models, which were developed in larvae instead of adult zebrafish, is probably related to the fact that at 7 dpf, the skin still appears capable of satisfying the oxygen requirements of the larvae[5]. The high resistance of zebrafish larvae to death by CPO allowed the expression of phenotypes with different grades of severity without the need to protect the larvae with antidotes. In the present manuscript, zebrafish OPP severity was graded as mild (grade 1), moderate (grade 2) and severe (grade 3) according to morphological and behavioural criteria. Further analyses at molecular, subcellular, cellular and tissue levels confirmed a progressive increase in the OPP severity from grade 1 to grade 3. Different scoring systems have been proposed for predicting outcome in human OPP, including the Namba scale of poisoning, poisoning severity score, Glasgow coma scale, acute physiology and chronic health evaluation II and predicted mortality rate[6, 7]. These systems are primarily based on the evaluation of clinical effects and mortality. Although some clinical signs and symptoms used in the

human scoring system cannot be analysed in zebrafish, most of the criteria used in the present study for grading severity are also used in humans. Thus, similar to the Namba scale[6] used in human OPP, a progressive increase in the severity of the motor problems and mortality from mild to severe poisoning was observed and correlated with a decrease in the cholinesterase activity. Our data show that the grading system used in the present manuscript is highly effective for predicting outcome (mortality and severity of the pathological effects at different levels of organization) in zebrafish OPP, strongly suggesting that even considering the differences in some analysed endpoints, this grading system is convenient for developing zebrafish models of human OPP with varying degrees of severity.

One of the most consistent effects we have found among the different grades of OPP is on the visual system. The cholinergic system plays a pivotal role in the physiology and development of a normal mammalian retina, where AChE is expressed very early[8]. When eyes from human volunteers were exposed to the AChE inhibitors sarin or physostigmine, different parameters of the visual function were altered, including sensitivity of the retina to light stimulus[9, 10], and retinal detachment has been noted as a complication in the treatment of glaucoma with some AChE inhibitors used in human pharmacology[11]. Although the potential retinotoxic effect of OP compounds on humans is still under discussion, chlorpyrifos has been reported to be highly toxic for the retina in different mammalian models[12, 13]. Consistently, grade 1 larvae exhibited impaired visual function, whereas retinotoxicity was found in grade 2 and 3 larvae.

The molecular initiating event (MIE) in the adverse outcome pathways resulting in OPP is AChE inhibition[14]. We have demonstrated that AChE inhibition is also the

MIE in our zebrafish models for OPP. Thus, a significant correlation was found between AChE inhibition and VMR in grade 1 larvae, and a full rescue of the phenotype was obtained when larvae were co-exposed with CPO and 2-PAM in grades 2 and 3. Moreover, some of the clinical features found in our models are typical of human cholinergic overstimulation, such as initial convulsion, paralysis or visual impairment. Moreover, overinflation of the swim bladder, which was found in grade 2 larvae, is clear evidence of cholinergic hyperstimulation. The swim bladder is an organ receiving cholinergic innervation from the vagus nerve, and this input seems to be involved in the processes of secreting gas into the swim bladder and contraction of the muscular sphincter closing the oesophageal end of the pneumatic duct[15]. Thus, an increase in the levels of ACh levels in the swim bladder cholinergic synapses should result an increase in the volume of gas stored inside this organ, which is the one of the features of our grade 2 phenotype.

Hypercontracture of the axial muscle fibres is the primary morphological feature of grade 2 larvae. Although our results demonstrated that hypercontracture was not caused by a mechanism resulting from depletion of ATP levels, a bioenergetic problem at the sarcomere level cannot be ruled out. Indeed, in the muscle fibres, mitochondrial ATP synthesis is coupled to phosphocreatine (PCr) synthesis by sarcomeric mitochondrial creatine kinase (mtCK)[16], and PCr, not ATP, is the high-energy phosphate essential for the relaxation process. In fact, under low PCr conditions, rigor-type hypercontracture develops even in the presence of mM concentrations of ATP[17]. Interestingly, the analysis of the transcriptional profile of grade 2 larvae showed that *ckmt2a*, a mitochondrial CK specifically expressed in the sarcomeres of zebrafish axial muscles, was one of the most strongly down-regulated genes, with -2.38-fold change (FDR: 1.75×10^{-6}), suggesting that a

reduction in the sarcomeric mtCK activity resulting in the depletion of PCr stores might be the mechanism producing the hypercontracture.

Release of glutamate and activation of NMDA receptors play essential roles in seizure activity and neurodegeneration following OPP by highly potent OP compounds in a mammalian model[18, 19]. Zebrafish larvae has been recently proposed as a useful model to elucidate the behavioural and molecular neuropharmacology of NMDA receptor antagonists[20], and NMDA receptor antagonists such as memantine or dizocilpine mateate (MK-801), which are used in biomedical research, exhibit similar effects on zebrafish[21, 22]. In the present study, we used memantine to analyse the potential involvement of NMDA receptor activation in the development of the grade 3 phenotype. Blocking NMDA receptors with memantine resulted in an increase in survival and a strong decrease in the prevalence of this phenotype. These data, along the transcriptomic data on calcium dysregulation and activation of inflammatory and immune responses, support common mechanisms of toxicity of OP compounds between mammals and zebrafish.

GSH is not only one of the most important antioxidants but also involved in other essential biological processes including xenobiotic detoxification. In fact, GSH seems to play an essential role in CPO metabolism, and different GSH conjugates, such as GSCPO, have been identified in the metabolism of this compound[23, 24]. The fact that the GSH precursor N-acetycysteine, but not any other antioxidant tested, decreased the severity of OPP in zebrafish suggests that this action might be mediated by an increase in the metabolism (GSH-conjugation) of CPO and not by an antioxidant action. Further studies, including additional OPs with different metabolic pathways, will be required to test this hypothesis.

Supplementary Figures

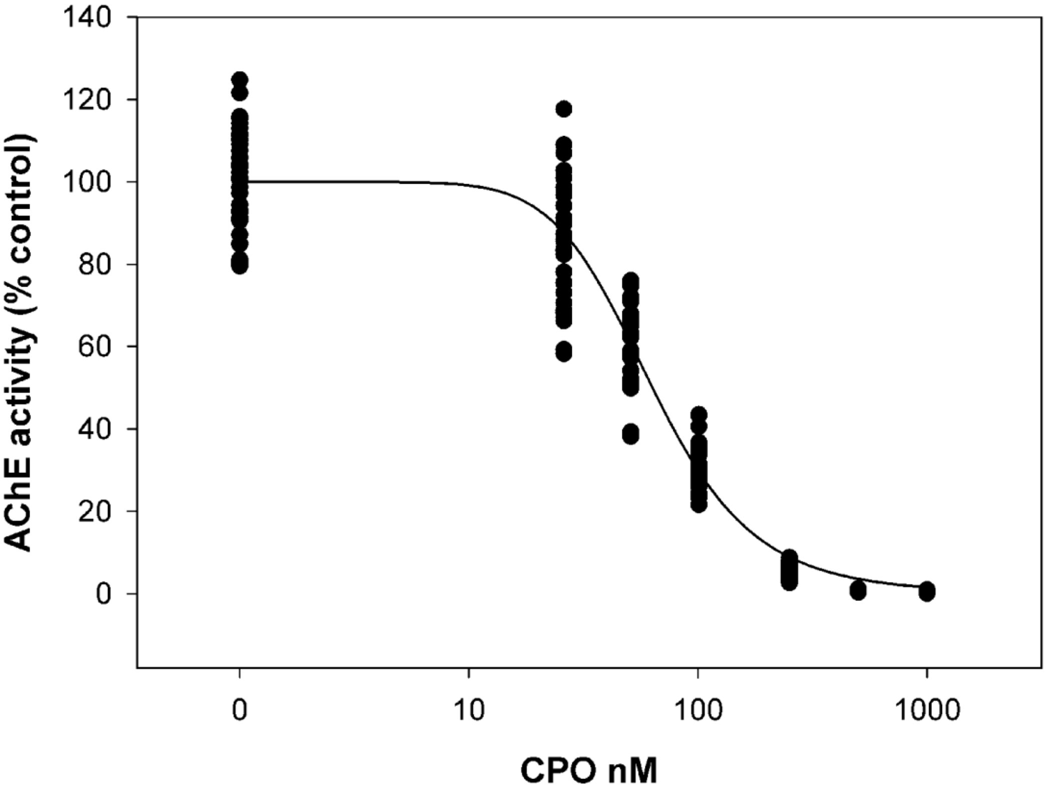

Supplementary Figure S1: Concentration-response analysis of AChE activity in zebrafish larvae exposed for 1 h to chlorpyrifos-oxon. Effect of chlorpyrifos-oxon (CPO) on AChE activity in 7 days post-fertilization larvae was extremely severe as early as 1 h exposure. Thus, at that time, an inhibition of AChE activity higher than 99% was found in larvae treated with 0.5 μM CPO. Regression parameters according to Eq.1 of fitted models to AChE inhibition were: IC50: 64.34 nM CPO/ s.e.m: 1.44/ $F(1,228)$/ $p<0.0001$/ r^2: 0.955/ n=229 (11-14 larvae for each treatment).

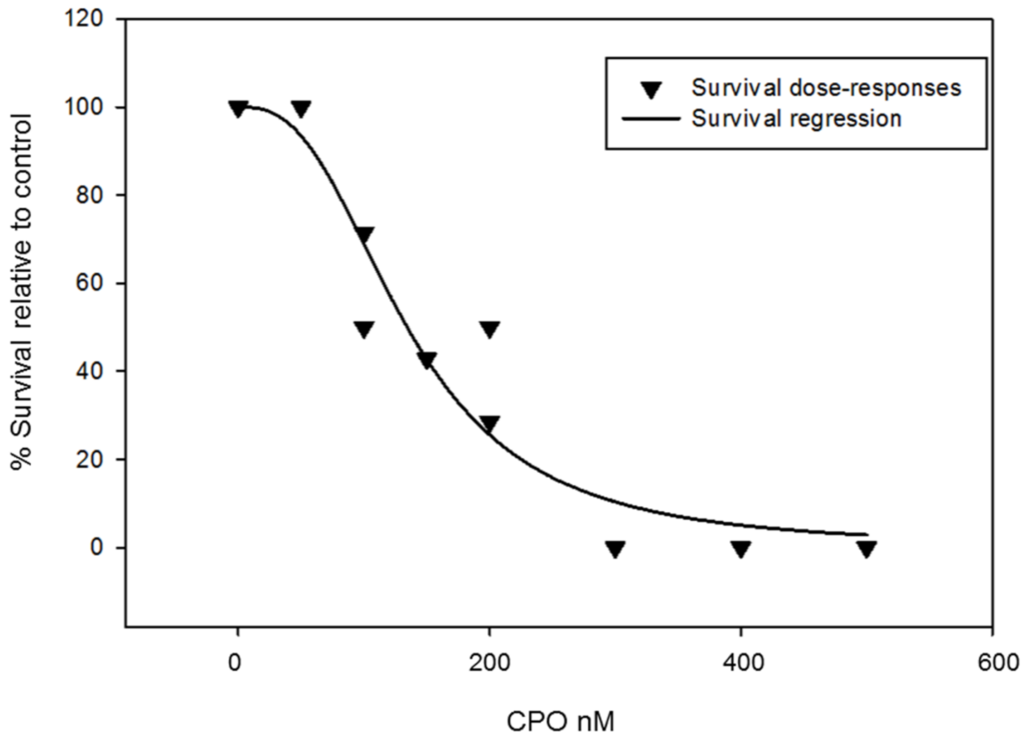

Supplementary Figure S2: Concentration-response analysis of the effect of 24 h exposure to chlorpyrifos-oxon on the survival of adult zebrafish. Regression parameters according to Eq.1 of fitted models to mortality responses were: LC50: 0.1344 μM CPO/ s.e.m: 0.009/ $p<0.0001$/ r^2: 0.947/ n=80.

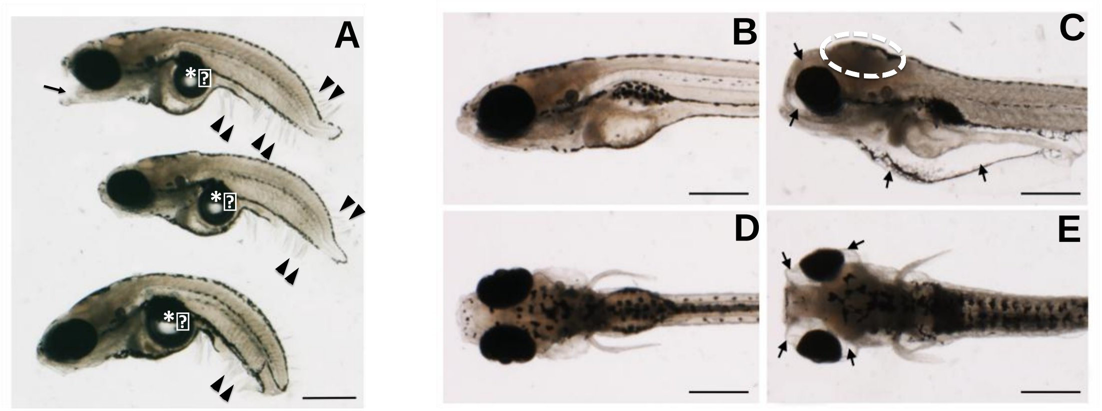

Supplementary Figure S3: **Morphological features on 8 days post-fertilization zebrafish larvae exhibiting moderate and severe acute organophosphorus poisoning by 24 h exposure to chlorpyrifos-oxon. (A)** Lateral views of three representative larvae exhibiting grade 2 (moderate) phenotype, with stiffness, impaired relaxation of the axial muscle fibers and overinflation of the swim bladder. Arrowheads indicate folding of the apical ectodermal ridge. White asterisks indicate the over-inflated swim bladder. Arrow indicates altered jaw in a larvae resulting from the hyperactivity of the jaw muscles. **(B-E)** Compacted head and periocular and/or peritoneal edema are common features in grade 3 (severe) phenotype. Lateral view (**B,C**) and dorsal view (**D,E**) of control (**B,D**) and grade 3 (**C,E**) larvae. Dashed trace shows the dramatic change in the morphology of the head in grade 3 larva (**C**). Presence of periocular (**C,E**) and peritoneal (**C**) edema are indicated by arrows. Scale bar: 400 μm.

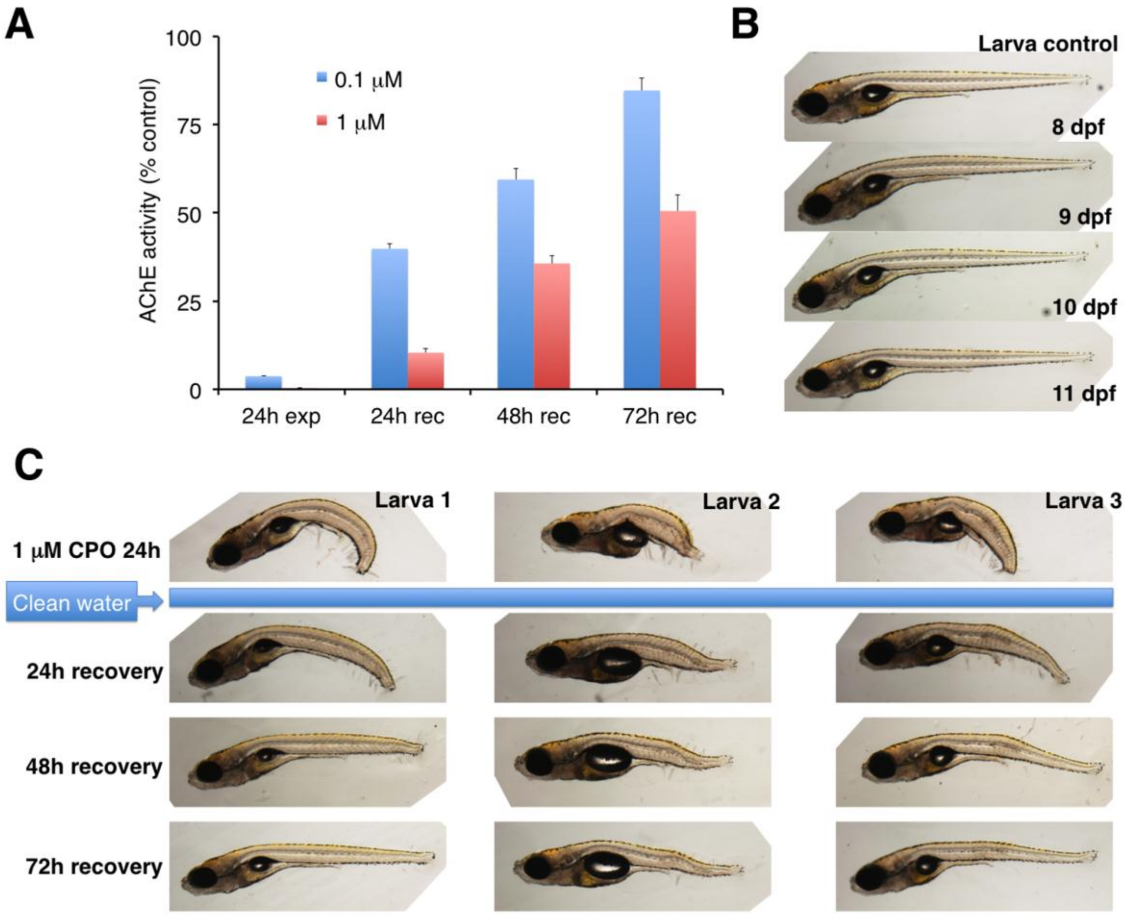

Supplementary Figure S4: Partial recovery of AChE activity and grade 2 phenotype in zebrafish larvae exposed to 1 μM chlorpyrifos-oxon after the transfer to clean water. **(A)** Analysis of the recovery of AChE activity shows a clear dependence on the grade of severity/chlorpyrifos-oxon (CPO) concentration. Larvae were exposed to 0.1 and 1 μM CPO for 24h and the resulting grades 1 (0.1 μM) and 2 (1 μM) were analysed (total n=192, with 16 individuals for each experimental group, mean ± s.e.m.). **(B)** Normal phenotype of a vehicle exposed 8-11 dpf zebrafish larva. **(C)** Phenotype of three grade 2 larvae immediately after 24 h exposure to 1 μM CPO, and 24-72 h after the transfer to clean water. A partial rescue of the phenotype is evident 3 days after the transfer to clean water.

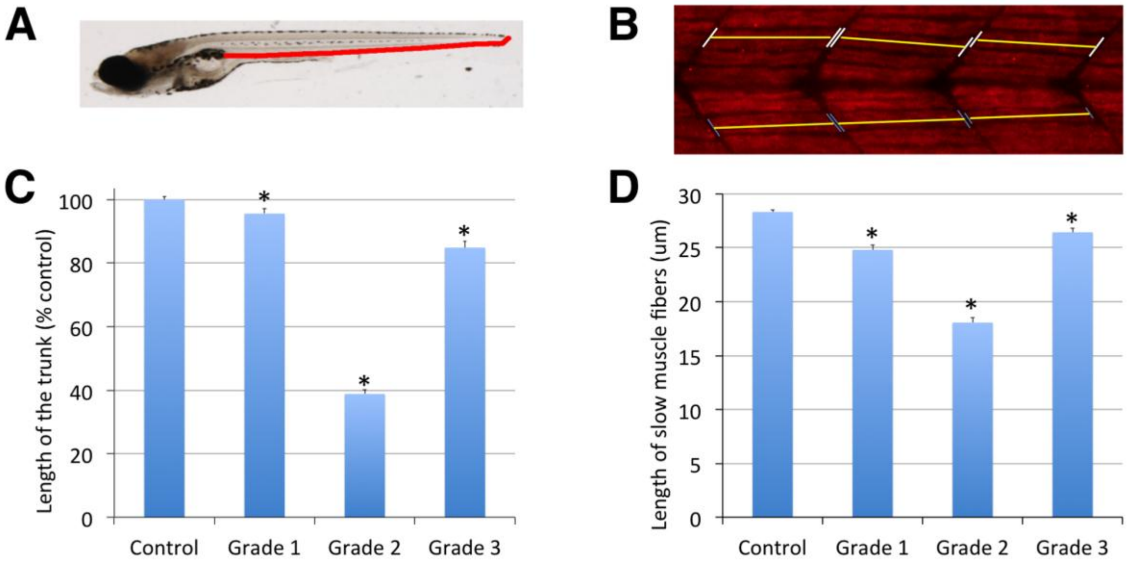

Supplementary Figure S5. Chlorpyrifos-oxon induces reduction in the length of the trunk and axial slow muscle fibers in zebrafish larvae. (A) Lateral view of a control 8 dpf zebrafish larva. Red segment, between the most caudal part of swim bladder and the tip of the tail, corresponds with the "length of the trunk", the part of the larva containing most of the axial muscle fibers. **(B)** The length of six slow muscle fibers, at the level of the lower intestine, up and down of the horizontal myoseptum, was measured in larvae control, as well as in larvae exposed for 24 h to 0.1 μM (grade 1), 0.75 μM (grade 2) and 6 μM (grade 3) chlorpyrifos-oxon (5 larvae per experimental group). In order to identify easily slow muscle fibers, whole-mount immunofluorescence analysis was performed by using a primary antibody against myosin heavy chain (F59, from DSHB). Length of the trunk and axial muscle fibers was determined by using the free image analysis software ImageJ (U. S. National Institutes of Health, Bethesda, Maryland, USA, http://imagej.nih.gov/ij/). **(C)** Length of the trunk was significantly reduced in the all three grades of severity, but specially in grade 2 (n=14-23; $p<0.005$ for grade 1 and $P<0.001$ for grade 2 and 3, mean ± s.e.m., one-way ANOVA with Dunnett's multiple comparison test). **(D)** Length of the slow muscle fibers was significantly reduced in all the grades, but specially in grade 2 larvae (mean ± s.e.m., $p<0.001$, one-way ANOVA with Dunnett's multiple comparison test).

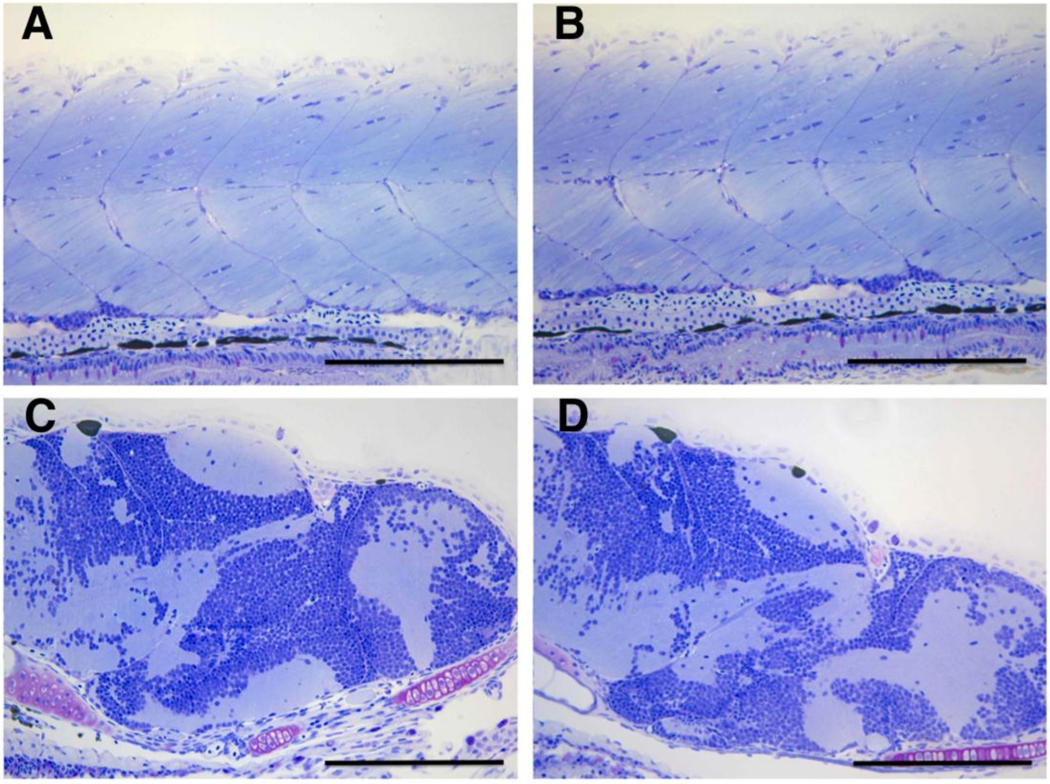

Supplementary Figure S6: Histopathological analysis of grade 1 phenotype. Semithin sections, at the trunk (**A,B**) and brain (**C,D**) level, of representative 8 days post-fertilization control (**A,C**) and grade 1 (**B,D**) larvae. No differences in the axial fast-twitch muscle fibers or brain are evident at histological level. Scale bar: 200 μm.

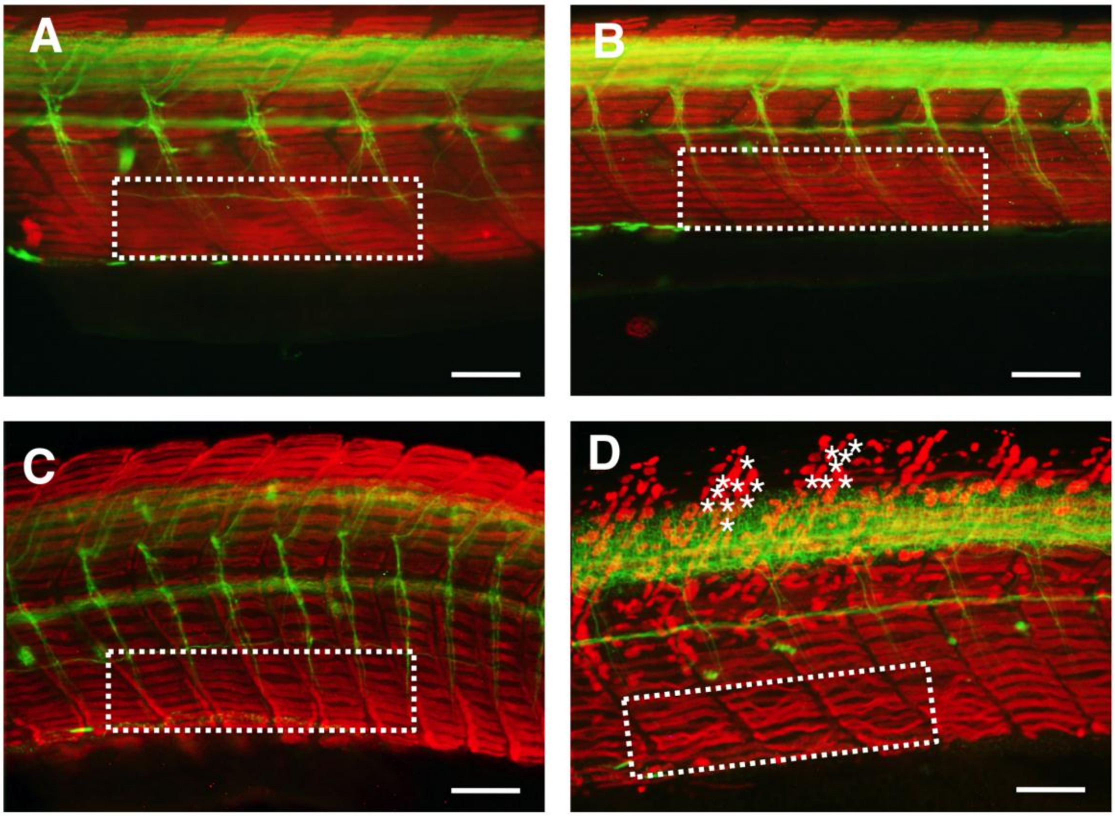

Supplementary Figure S7: Characterization of pathological features at the trunk level in the different grades of severity of acute organophosphorus poisoning induced by chlorpyrifos-oxon in zebrafish larvae. Lateral view of the trunk of 8 dpf zebrafish larvae control (**A**), grade 1 (**B**), grade 2 (**C**) and grade 3 (**D**) after the double whole-mount immunofluorescence with F59 (red staining) and anti-acetylated alpha tubulin (green staining) antibodies. This labelling provides information on integrity and architecture of slow muscle fibers and spinal axonal tracts, respectively. The area within the white dashed lines shows the arrangement of the slow muscle fibers in a similar region of the ventral trunk in the different phenotypes. Asterisks indicate rupture or detachments of slow muscle fibers along the dorsal trunk of a representative grade 3 larva. Lateral view of the trunk (dorsal is to the top and anterior is to the left). Scale bar: 100 μm.

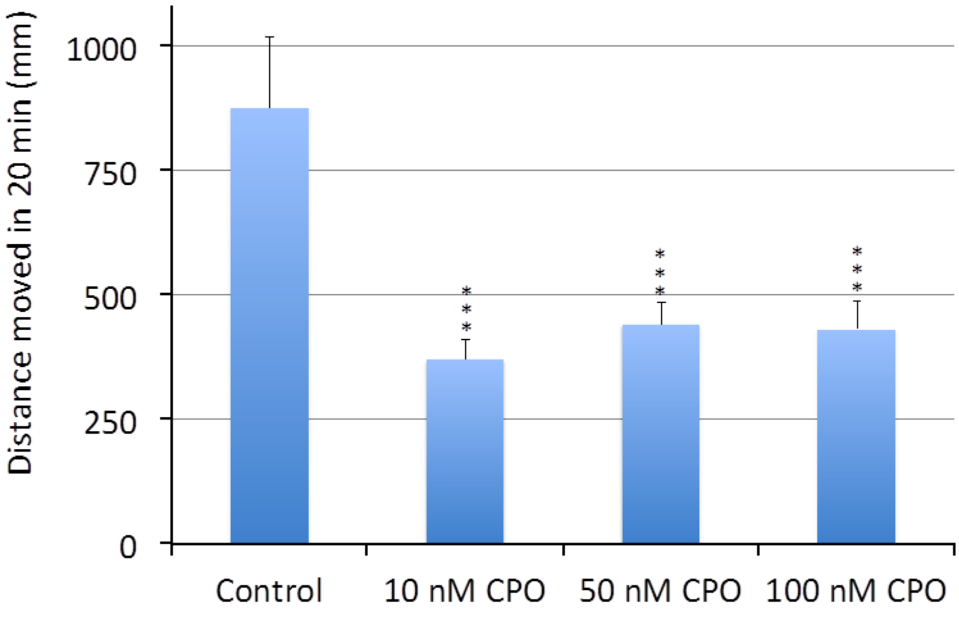

Supplementary Figure S8. Basal locomotor activity was analyzed by measuring the distance moved by the 8 days post-fertilization larvae in 20 min. A significant reduction (one-way ANOVA with Dunnett's multiple comparison test, $F(3,52) = 8.829$, $p<0.0001$) in basal motor activity was found in grade 1 larvae exposed for 24 h to 10 nM (370.3 ± 41.4 mm, mean ± s.e.m., n=16), 50 nM (441.9 ± 46.0 mm, mean ± s.e.m., n=15) and 100 nM CPO (430.8 ± 58.4 mm, mean ± s.e.m., n=13) compared to the control (875.7 ± 142.2 mm, mean ± s.e.m., n=12).

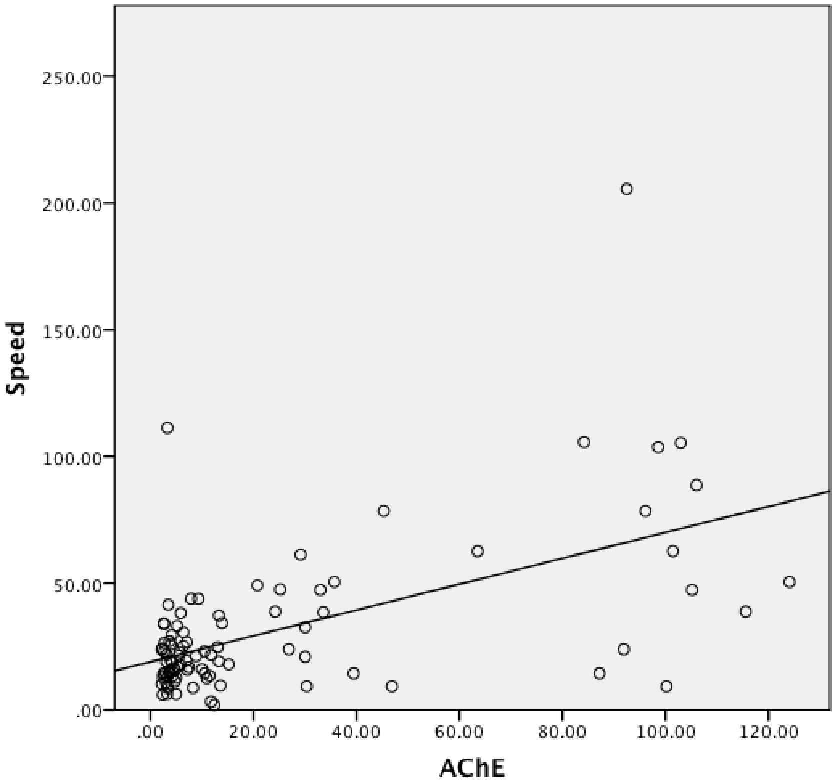

Supplementary Figure S9. Decrease in the Visual Motor Response (VMR) in individual grade 1 larvae is significantly correlated to the acetylcholinesterase (AChE) activity in the same larvae. At 7 days post-fertilization, zebrafish larvae were exposed for 24 h to the solvent (0.1% DMSO) or different concentrations of chlorpyrifos-oxon (10 nM-100 nM). Then, VMR and AChE activity were sequentially analysed on the same individual larvae. A significant correlation was found between these two parameters in the treated larvae (Pearson correlation, $r^2 = 0.323$, $p<0.001$, n=92).

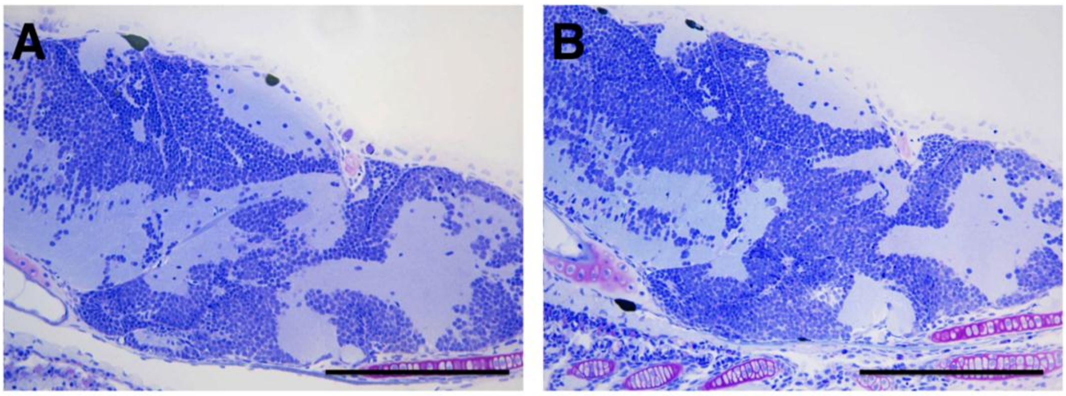

Supplementary Figure S10. Histophathological evaluation of the central nervous system in grade 2 phenotype. Semithin section of the brain of representative control (**A**) and grade 2 (**B**) 8 days post-fertilization larvae. No differences in the histology of the brain were evident in grade 2 larvae respect to the control. Scale bar: 200 μm.

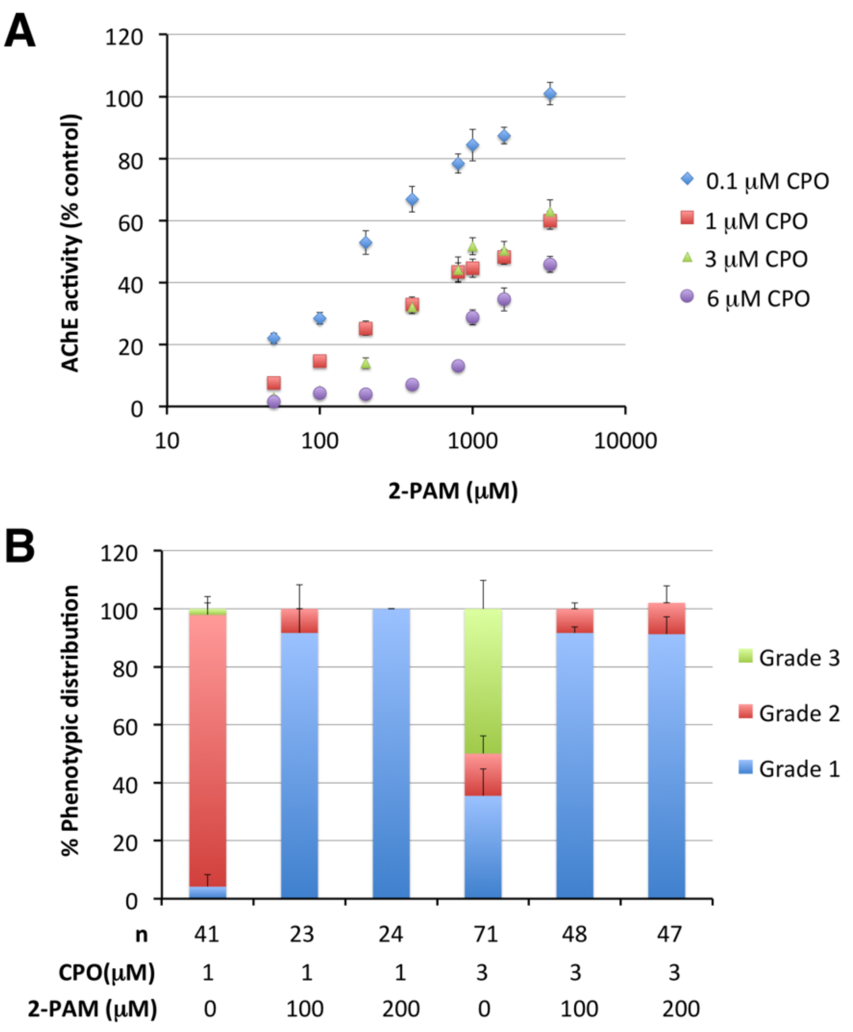

Supplementary Figure S11: Pralidoxime recovers both acetylcholinesterase activity and phenotype in 8 days post-fertilization zebrafish larvae exposed for 24 h to different chlorpyrifos-oxon concentrations. (**A**) The degree of recovery of acetylcholinesterase (AChE) activity is related with the concentrations of both chlorpyrifos-oxon (CPO) and pralidoxime (2-PAM). Full recovery of AChE activity only was achieved with the combination of the lowest CPO (0.1 µM CPO) and highest 2-PAM (3,200 µM) concentrations. Data presented as mean ± s.e.m (n = 16 for each experimental group). (**B**) Effect of 2-PAM on grade 2 and 3 phenotypes. Grade 2 larvae, generated when 7 days post-fertilization (dpf) larvae were exposed to 1 µM CPO for 24 h, were fully rescued by co-exposure with 200 µM 2-PAM. Similarly, grade 3 larvae generated by exposure to 3 µM CPO were fully rescued by co-exposure to 200 µM 2-PAM. Values represent averages from 3-4 independent biological samples (8-20 larvae each). Data presented as mean ± s.e.m.

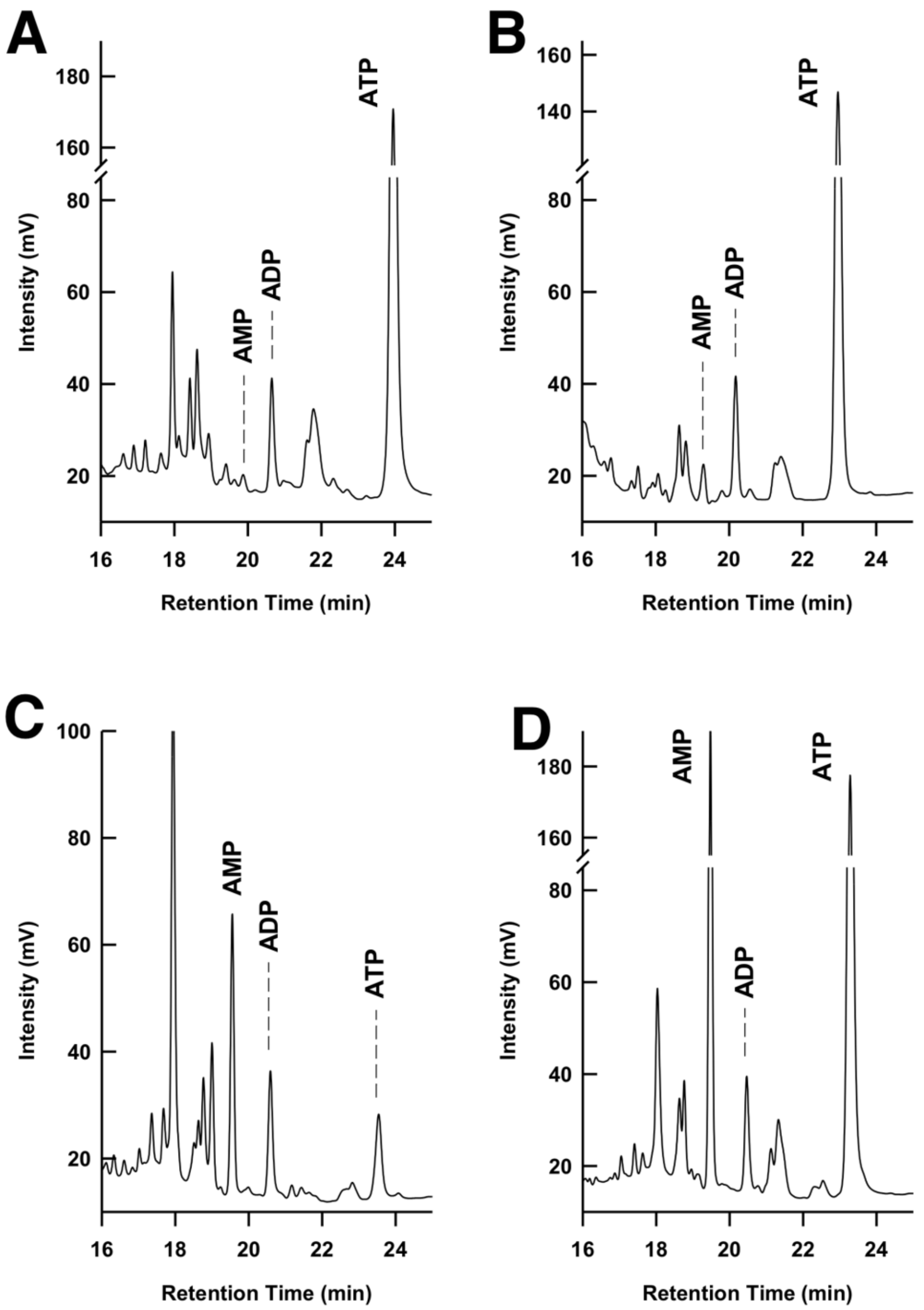

Supplementary Figure S12: Determination of AMP, ADP and ATP in 8 days post-fertilization zebrafish larvae homogenates by HPLC. The figure shows representative chromatograms identifying adenine nucleotide peaks in (**A**) control larvae, (**B**) grade 3 larvae, (**C**) larvae treated for 24h with 40 mM 2-DOG and then for 2h with 5 uM oligomycin plus 40 mM 2-DOG and (**D**) extracts from control larvae supplemented with AMP to confirm the retention time of this nucleotide.

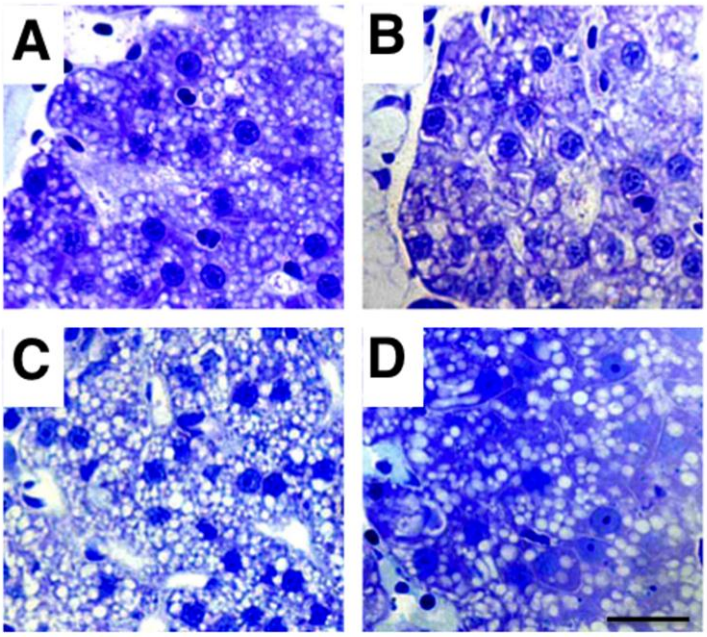

Supplementary Figure S13: Liver structure is not altered in acute organophosphorus poisoning in zebrafish. Normal hepatic structures and normal hepatocytes are observed both in 8 days post-fertilization control **(A),** grade 1 **(B)**, grade 2 **(C)** and grade 3 **(D)** zebrafish larvae. Hepatocyte nuclear size may not be homogeneous in the same fish and darker nuclei in **D** correspond to erythroblasts/erythrocytes. The presence of lipid droplets (white spherical structures) is a normal observation corresponding to yolk remnants, and their number may not always be homogeneous in fish from the same batch. Scale bar: 80 μm.

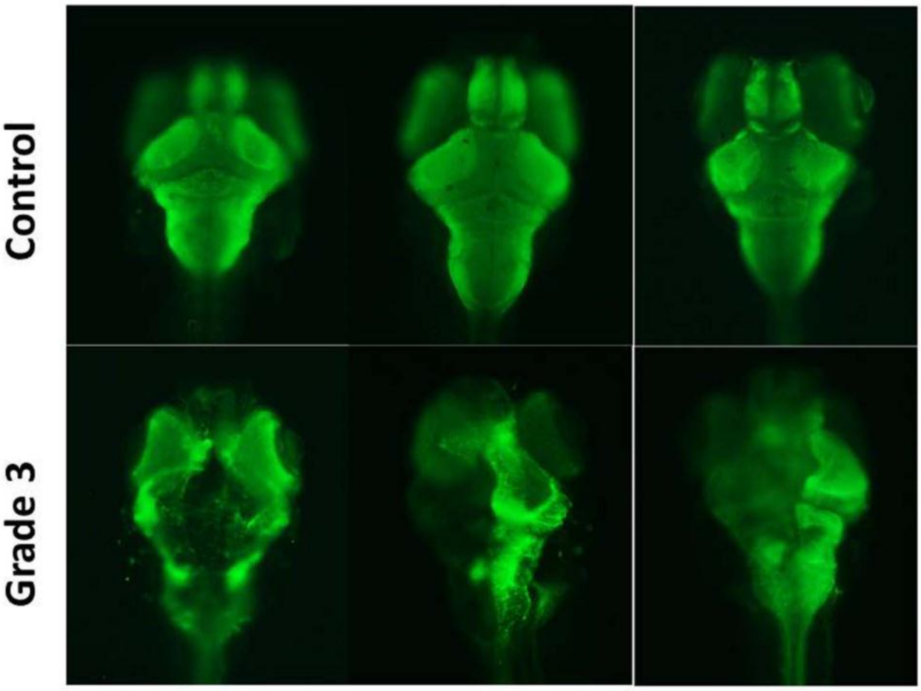

Supplementary Figure S14: Grade 3 larvae exhibit severe impairment in the brain architecture. Dorsal view of 3 heads of 8 days post-fertilization control and grade 3 zebrafish larvae, after the staining of the axonal tracts with an acetylated alpha-tubulin (green) monoclonal antibody by whole-mount immunofluorescence. The severe damage in the axonal tracts found in the brain of grade 3 larvae is consistent with the results of the semithin sections and transmission electron microscopy (TEM).

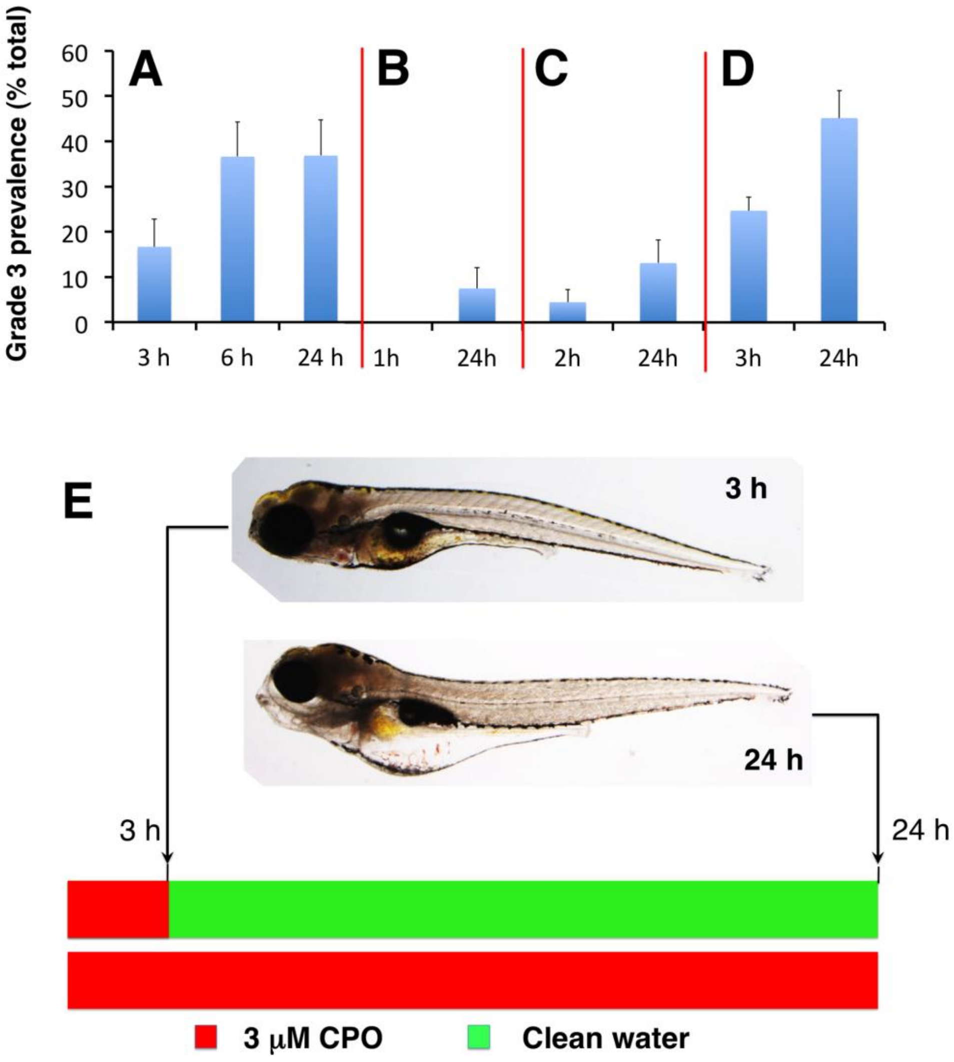

Supplementary Figure S15. Commitment to grade 3 phenotype occurs during the first 3 h post-exposure. (**A**) 7 dpf zebrafish larvae were exposed to 3 μM chlorpyrifos-oxon (CPO) and the prevalence of grade 3 phenotype was evaluated after 3, 6 and 24 h exposure. No differences en the grade 3 prevalence were found between larvae exposed to CPO for 6 and 24 h [n= 6 experimental groups with 8 larvae each; p=0.492, Student's t-test (t(10) = 0.0199)]. (**B-D**) 7 dpf zebrafish larvae were exposed to 3 μM CPO for 1 h (**B**), 2 h (**C**) and 3 h (**D**) and then transferred to clean water for additional 23, 22 and 21 h. For each experimental group, prevalence of grade 3 phenotype was analysed at the end of the CPO exposure period and after the period in clean water. At 8 dpf, no differences in grade 3 prevalence were found between the larvae continuously exposed to 3 μM CPO for 24 h and those exposed for only 3 h and then transferred to clean water for additional 21 h [n= 6 experimental groups with 8 larvae each; p=0.212,

Student's t-test ($t(10) = 0.836$)]. (**E**) Lateral view of a single representative zebrafish grade 3 larva exposed to 3 uM CPO for 3 h (top) and then transferred to clean water for additional 21 h (bottom). At 3 h are already evident the changes in the morphology of the head typical of grade 3 larvae. No recovery of the phenotype was found after 21 h of depuration in clean water (bottom). By contrast, the phenotype of the larvae after the depuration period was indistinguishable of the larvae exposed continuously during 24 h. Data presented as mean ± s.e.m.

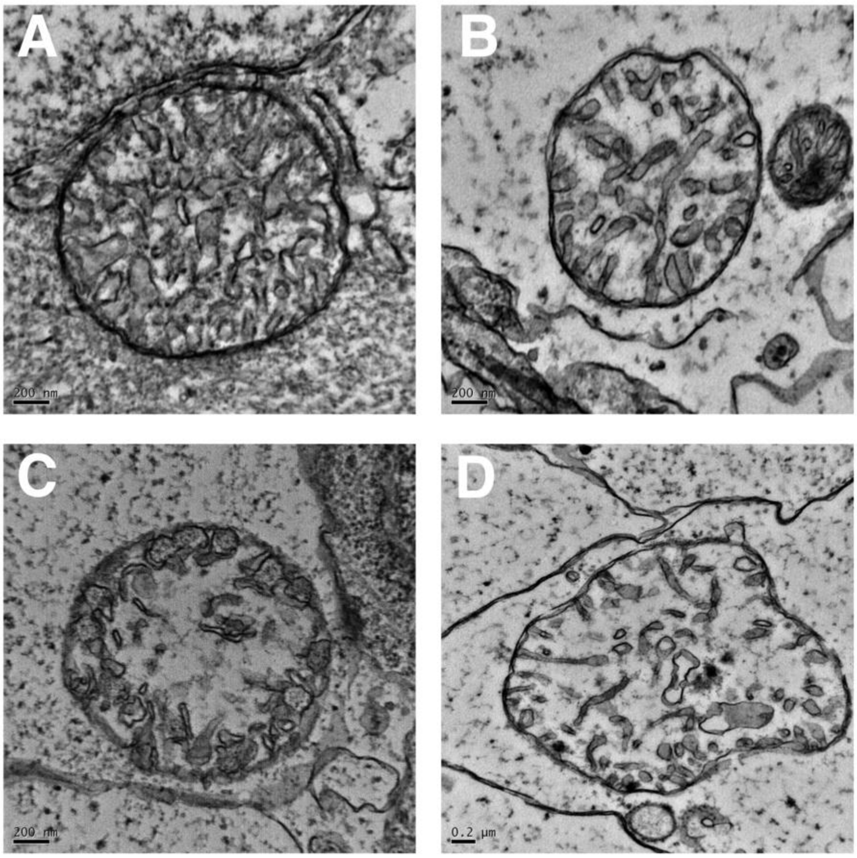

Supplementary Figure S16. Swelling and loss of cristae in the mitochondria of the grade 3 larvae (brain). Transmission electron microscopy images of one mitochondrion control (**A**) and three mitochondria from the brain of 8 days post-fertilization grade 3 larvae (**B-D**).

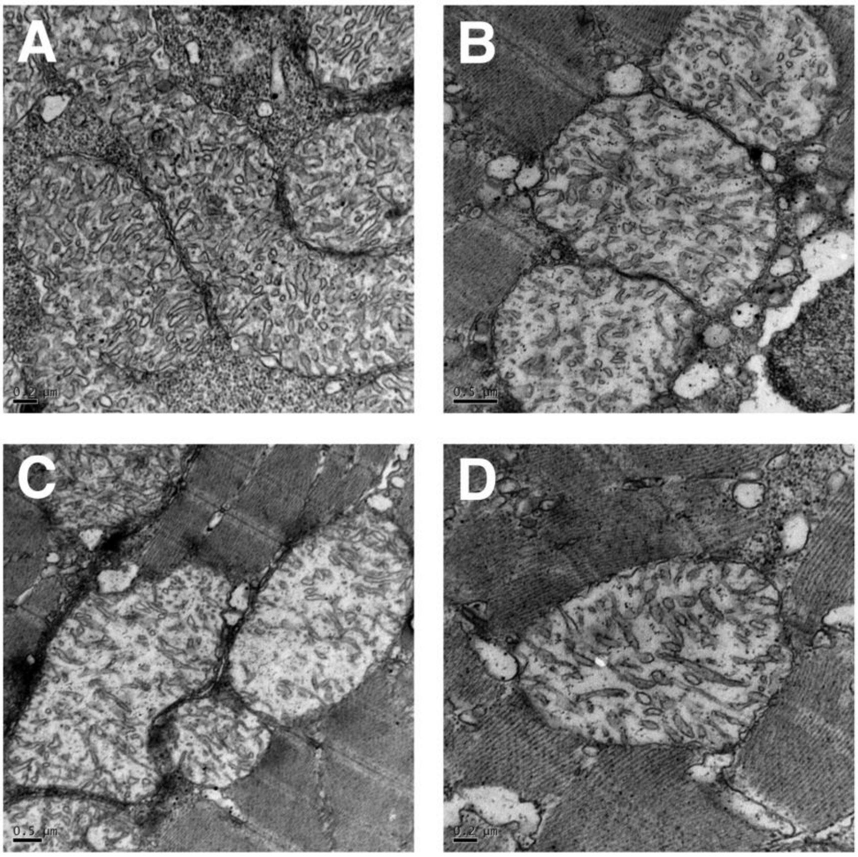

Supplementary Figure S17. Swelling and loss of cristae in the mitochondria of the grade 3 larvae (fast-twitch axial muscle fibers). Transmission electron microscopy images of one mitochondrion control (**A**) and three mitochondria from the fast muscle fibers of 8 days post-fertilization grade 3 larvae (**B-D**).

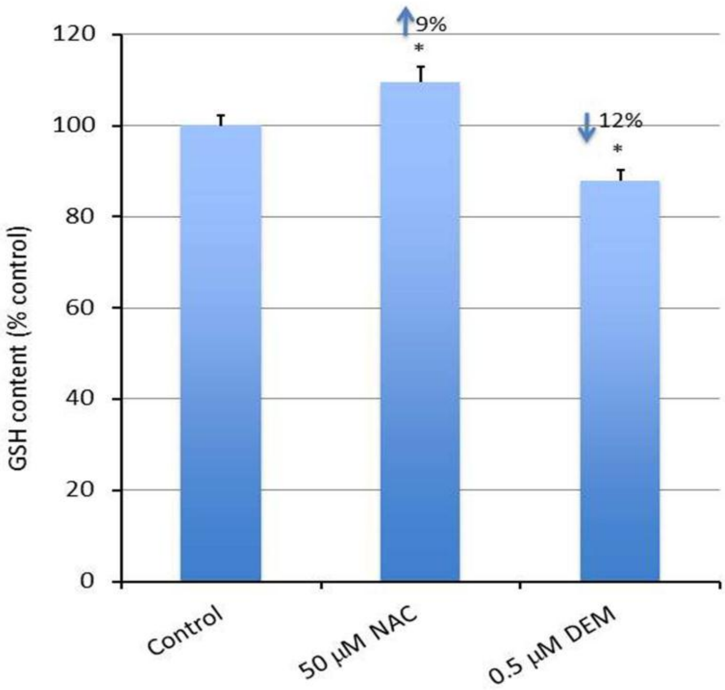

Supplementary Figure S18. Modulation of the endogenous levels of reduced glutathione in zebrafish larvae by using N-acetylcysteine and diethyl maleate. At 7 days post-fertilization, zebrafish larvae were exposed to the chemicals for 24 h. N-acetylcysteine (NAC) increased and dietyl maleate (DEM) decreased total glutathione (GSH) levels respect to the controls (*$p<0.05$, Student's t-test; NAC: $p=0.0265$, $t(8) = 2.266$; DEM, $p=0.023$, $t(13) = 2.203$). Data represent mean ± s.e.m. of 5 pools (NAC) or 8 pools (DEM), with 5 larvae each.

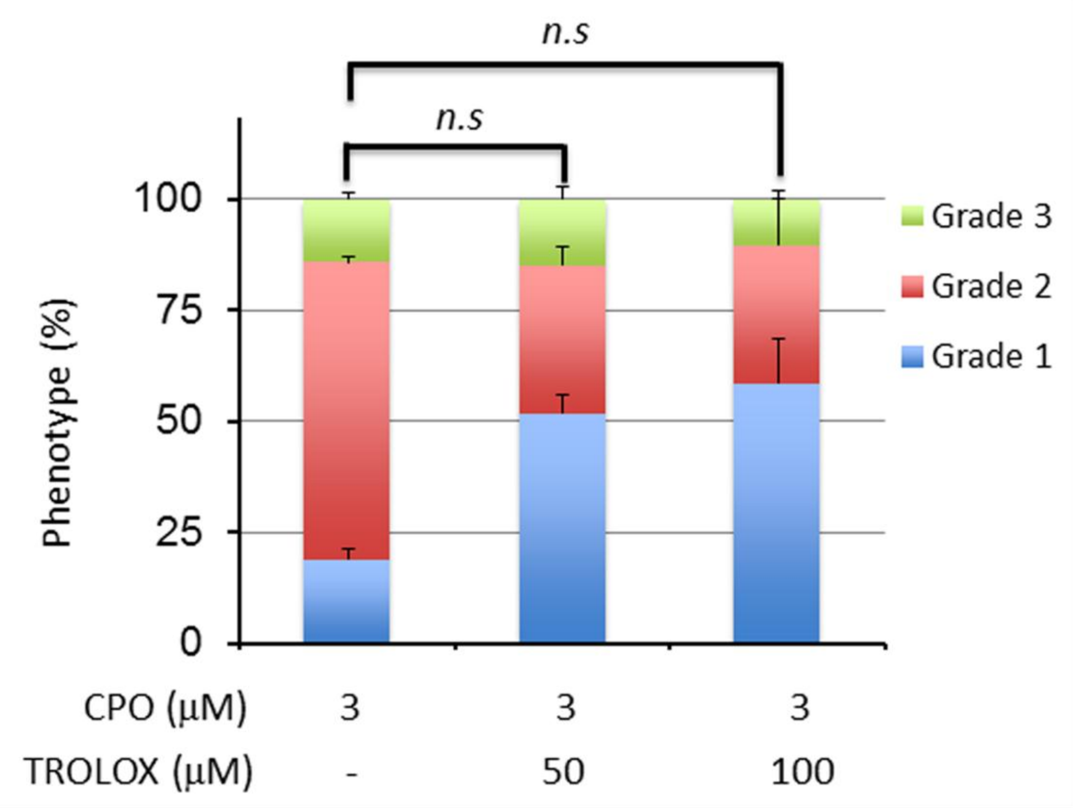

Supplementary Figure S19. **The antioxidant soluble vitamin E has not a significant effect on the prevalence of grade 3 phenotype.** Two different concentrations of vitamin E (TROLOX) were not able to decrease significantly the prevalence of grade 3 phenotype (4-5 pools per condition with 15-23 larvae each; p=0.421 / $t(6)$ = 0.208 and p= 0.0905 / $t(6)$ = 1.513, respectively, Student's t-test). 7 days post fertilization larvae were first pre-treated with either 50 or 100 µM of trolox for 3h and then co-exposed with 3 µM of CPO for 24h. Abbreviations: *n.s*, no significant

Supplementary Tables

Supplementary Table S1: Chlorpyrifos-oxon measured concentrations in fish water

Time 0			Time 24		
**Nominal concentration (uM)**	**Measured concentration (uM)**	**Mean measured concentration (uM)**	**Nominal concentration (uM)**	**Measured concentration (uM)**	**Mean measured concentration (uM)**
0.1	0.1018		0.1	0.0371	
0.1	0.9992	0.0996	0.1	0.0405	0.0382
0.1	0.0978		0.1	0.0370	
1	0.9809		1	0.6947	
1	0.9741	0.9955	1	0.6880	0.6840
1	1.0314		1	0.6693	
3	2.9598		3	2.0389	
3	3.0260	2.9894	3	1.8878	1.9924
3	2.9823		3	2.0504	

Chlorpyrifos-oxon concentration was measured in the water when the solutions were added (time 0) and after the incubation with the zebrafish larvae (time 24 h).

Supplementary Table S2. Results on bioenergetics state of control and 8 days post-fertilization grade 2 larvae

		Concentration (nM)								
		**ATP**	**ADP**	**AMP**	**ATP/ADP**	**ATP/AMP**	**Energy charge**	**% ATP**	**%ADP**	**%AMP**
	Pool 1	1270	115	9.4	11.1	137.5	0.95	91.1	8.2	0.66
	Pool 2	987	106	9.6	9.3	104.8	0.94	89.6	9.6	0.85
**Control**	Pool 3	961	103	10.4	9.4	93.7	0.94	89.5	9.6	0.95
	Pool 4	1044	121	8.8	8.7	121.1	0.94	89.0	10.3	0.73
	Pool 5	1304	131	14.8	10.0	89.1	0.94	90.0	9.0	1.01
	Average	1113.3	115.0	10.6	9.7	109.2	0.945	89.8	9.3	0.8
	SE	72.3	5.1	1.1	0.4	9.0	0.002	0.4	0.3	0.1
	Pool 1	1319	148	13.6	8.9	98.3	0.94	89.1	10.0	0.91
	Pool 2	1334	164	18.3	8.2	73.6	0.93	88.0	10.8	1.20
**Grade 2**	Pool 3	1266	147	14.9	8.6	85.7	0.94	88.7	10.3	1.04
	Pool 4	1363	102	11.8	13.4	116.4	0.96	92.3	6.9	0.79
	Pool 5	1335	100	11.1	13.4	121.8	0.96	92.3	6.9	0.76
	Average	1323	132	13.9	10.5	99.2	0.946	90.1	9.0	0.94
	SE	18	15	1.4	1.3	10.1	0.006	1.0	1.0	0.09

Supplementary Movie Legends

Supplementary Movie 1

Grade 2 larvae, generated by waterborne exposure to 1.5 μM chlorpyrifos-oxon for 24 h, became completely paralyzed, with touch-evoked escape response fully abolished.

Supplementary Movie 2

Grade 2 larvae, generated by waterborne exposure to 0.75 μM chlorpyrifos-oxon for 24 h, exhibited creeping, with increased pectoral fin and jaw movements. In contrast, the strong stiffness observed at the axial muscle fibers level prevent the movement of the trunk in this larva.

Supplementary References

1. Le Bihanic F, *et al*. Developmental toxicity of PAH mixture in fish early life stages. Part II: adverse effects in Japanese medaka. *Environ Sci Pollut Res Int* **21**, 13732-13743 (2014).
2. Carey JL, Dunn C, Gaspari RJ. Central respiratory failure during acute organophosphate poisoning. *Respir Physiol Neurobiol* **189**, 403-410 (2013).
3. Randall DJ, Taylor E. Evidence of a role for catecholamines in the control of breathing in fish. *Rev Fish Biol Fisheries* **1**, 139-157 (1991).
4. Bradbury S, Carlson R, Henry T, Padilla S, Cowden J. Toxic responses of the fish nervous system. In: *The Toxicology of Fishes, CRC Press, Taylor and Francis* (2008).
5. Rombough P. Gills are needed for ionoregulation before they are needed for O_2 uptake in developing zebrafish, Danio rerio. *J Exp Biol* **205**, 1787-1794 (2002).
6. Namba T, Nolte CT, Jackrel J, Grob D. Poisoning due to organophosphate insecticides. Acute and chronic manifestations. *Am J Med* **50**, 475-92 (1971).
7. Sam KG, *et al*. Poisoning severity score, APACHE II and GCS: effective clinical indices for estimating severity and predicting outcome of acute organophosphorus and carbamate poisoning. *J Forensic Leg Med* **16**, 239-47 (2009).

8. Bytyqi AH, *et al*. Impaired formation of the inner retina in an AChE knockout mouse results in degeneration of all photoreceptors. *Eur J Neurosci* **20**, 2953-2962 (2004).

9. Gazzard M, Thomas DP. A comparative study of central visual field changes induced by sarin vapour and physostigmine eye drops. *Exp Eye Res* **20**, 15-21 (1975).

10. Rubin LS, Goldberg MN. Effect of sarin on dark adaptation in man: threshold changes. *J Appl Physiol* **11**, 439-444 (1957).

11. Schuman JS. Antiglaucoma medications: a review of safety and tolerability issues related to their use. *Clin Ther* **22**, 167-208 (2000).

12. Yu F, *et al*. Apoptotic effect of organophosphorus insecticide chlorpyrifos on mouse retina in vivo via oxidative stress and protection of combination of vitamins C and E. *Exp Toxicol Pathol* **59**, 415-423 (2008).

13. Ahmed NS, Mohamed AS, Abdel-Wahhab MA. Chlorpyrifos-induced oxidative stress and histological changes in retinas and kidney in rats: Protective role of ascorbic acid and alpha tocopherol. *Pesticide Biochem Physiol* **98**, 33-38 (2010).

14. Pereira EF*, et al.* Animal models that best reproduce the clinical manifestations of human intoxication with organophosphorus compounds. *J Pharmacol Exp Ther* **350**, 313-321 (2014).

15. Robertson G, McGee C, Dumbarton T, Croll R, Smith F. Development of the swimbladder and its innervation in the zebrafish, Danio rerio. *J Morphol* **268**, 967-985 (2007).

16. Yaniv Y, Spurgeon HA, Ziman BD, Lakatta EG. Ca^{2+}/calmodulin-dependent protein kinase II (CaMKII) activity and sinoatrial nodal pacemaker cell energetics. *PloS one* **8**, e57079 (2013).

17. Ventura-Clapier R, Vassort G. Rigor tension during metabolic and ionic rises in resting tension in rat heart. *J Mol Cell Cardiol* **13**, 551-561 (1981).

18. Shih T-M, McDonough JH. Neurochemical Mechanisms in Soman-induced Seizures. *J Appl Toxicol* **17**, 255-264 (1997).

19. Solberg Y, Belkin M. The role of excitotoxicity in organophosphorous nerve agents central poisoning. *Pharmacol Sci* **18**, 183-185 (1997).

20. Chen J, Patel R, Friedman TC, Jones KS. The behavioral and pharmacological actions of NMDA receptor antagonism are conserved in zebrafish larvae. *Int J Comp Psychol* **23**, 82-90 (2010).

21. Feldman B, Tuchman M, Caldovic L. A zebrafish model of hyperammonemia. *Mol Genet Metab* **113**, 142-147 (2014).

22. Best JD*, et al.* Non-associative learning in larval zebrafish. *Neuropsychopharmacology* **33**, 1206-1215 (2008).

23. Fujioka K, Casida JE. Glutathione S-Transferase conjugation of organophosphorus pesticides yields S-Phospho-, S-Aryl-, and S-Alkylglutathione derivatives. *Chem Res Toxicol* **20**, 1211-1217 (2007).

24. Choi K, Joo H, Rose RL, Hodgso E. Metabolism of chlorpyrifos and chlorpyrifos oxon by human hepatocytes. *J Biochem Mol Toxicol* **20**, 279-291 (2006).
